# Microbe-associated molecular pattern recognition receptors have little effect on endophytic *Arabidopsis thaliana* microbiome assembly in the field

**DOI:** 10.3389/fpls.2023.1276472

**Published:** 2023-11-08

**Authors:** Caroline Oldstone-Jackson, Feng Huang, Joy Bergelson

**Affiliations:** ^1^ Department of Ecology and Evolution, The University of Chicago, Chicago, IL, United States; ^2^ Plant Protection Research Institute, Guangdong Academy of Agricultural Sciences, Guangzhou, Guangdong, China; ^3^ Center for Genomics and Systems Biology, Department of Biology, College of Arts and Science, New York University, New York, NY, United States

**Keywords:** microbiome, microbial diversity, microbial communities, plant immunity, microbe-associated molecular pattern, MAMP, Arabidopsis, field

## Abstract

Plant microbiome structure affects plant health and productivity. A limited subset of environmental microbes successfully establishes within plant tissues, but the forces underlying this selectivity remain poorly characterized. Transmembrane pattern recognition receptors (PRRs), used by plants to detect microbe-associated molecular patterns (MAMPs), are strong candidates for achieving this selectivity because PRRs can potentially interact with many members of the microbiome. Indeed, MAMPs found in many microbial taxa, including beneficials and commensals, can instigate a robust immune response that affects microbial growth. Surprisingly, we found that MAMP-detecting PRRs have little effect on endophytic bacterial and fungal microbiome structure in the field. We compared the microbiomes of four PRR knockout lines of *Arabidopsis thaliana* to wild-type plants in multiple tissue types over several developmental stages and detected only subtle shifts in fungal, but not bacterial, *β*-diversity in one of the four PRR mutants. In one developmental stage, *lore* mutants had slightly altered fungal *β*-diversity, indicating that LORE may be involved in plant-fungal interactions in addition to its known role in detecting certain bacterial lipids. No other effects of PRRs on *α*-diversity, microbiome variability, within-individual homogeneity, or microbial load were found. The general lack of effect suggests that individual MAMP-detecting PRRs are not critical in shaping the endophytic plant microbiome. Rather, we suggest that MAMP-detecting PRRs must either act in concert and/or are individually maintained through pleiotropic effects or interactions with coevolved mutualists or pathogens. Although unexpected, these results offer insights into the role of MAMP-detecting PRRs in plant-microbe interactions and help direct future efforts to uncover host genetic elements that control plant microbiome assembly.

## 1 Introduction

Plants closely associate with complex microbial communities composed of bacteria, fungi, oomycetes, and other microorganisms. This community, or microbiome, colonizes the soil surrounding the roots (rhizosphere), external plant surfaces, and the spaces within plants (endosphere). The microbiome affects plant growth (Vessey, 2003), phenology (Lau and Lennon, 2011; Wagner et al., 2014), abiotic stress tolerance (Rodriguez et al., 2008) and disease resistance (Vannier et al., 2019). These observations have sparked a major effort to engineer plant microbiomes to improve crop yields and tolerance to abiotic and biotic stress, thus reducing dependency on chemical fertilizers and pesticides and increasing crop resiliency to the mounting challenges of climate change. To harness the microbiome to achieve these agricultural goals, the rules governing plant microbiome assembly processes must be elucidated.

Plant microbiomes are primarily composed of microbes derived from the environment. Only a subset of environmental microbes associate with plants (Bulgarelli et al., 2012; Lundberg et al., 2012; Vorholt, 2012). Numerous factors, including abiotic conditions, microbe-microbe interactions, and host-effects, underpin this selective filtering (Fitzpatrick et al., 2020). Selectivity typically increases in the endosphere; the microbial communities within plant tissues are generally less diverse than those of external plant surfaces (Bulgarelli et al., 2012; Lundberg et al., 2012; Bodenhausen et al., 2013; Coleman-Derr et al., 2016; Chen et al., 2020; Mina et al., 2020). This filtering effect is also tissue-specific (Beilsmith et al., 2021). Host genetics likely play a role in filtering environmental microbes, as plant microbiomes are typically more similar within species than between species, even when grown in common environments (Naylor et al., 2017; Tkacz et al., 2020; Wippel et al., 2021). Within-species genotype can also affect microbiome composition (Bulgarelli et al., 2012; Lundberg et al., 2012; Horton et al., 2014; Brachi et al., 2022).

How are microbes filtered from the environment during colonization of plant tissues? The plant immune system is an obvious candidate, as host-microbe interactions often involve the host’s immune system. The plant immune system recognizes non-self and modified-self molecules via two main classes of receptor proteins (Jones and Dangl, 2006; Dodds and Rathjen, 2010). One class, transmembrane pattern recognition receptors (PRRs), detects microbe-associated molecular patterns (MAMPs) and endogenous signals caused by damage to plant cells, known as damage-associated molecular patterns (DAMPs). MAMPs are non-self molecules commonly found across broad taxonomic classes of microbes that contribute to microbial fitness in numerous environments, such as flagellin, elongation factor Tu, chitin, peptidoglycan, and lipid metabolites (Boller and Felix, 2009; Tang et al., 2017; Kutschera et al., 2019; Schellenberger et al., 2021). Indeed, genomic surveys reveal that most, if not all, plant-associated bacteria produce immunogenic MAMPs (Garrido-Oter et al., 2018; Teixeira et al., 2019). In addition to PRRs, plants employ Resistance (R) receptors to detect effectors, molecules secreted by microbes to suppress plant immunity and/or manipulate the plant environment to promote microbial growth (Cui et al., 2015). R proteins can also detect the modified plant targets of these effectors.

Upon detection of MAMPs, plants respond with a multifaceted response including ion fluxes, reactive oxygen species bursts, and massive transcriptional reprogramming (Boller and Felix, 2009; Macho and Zipfel, 2014). This response, pattern-triggered immunity (PTI), moderates pathogen growth and is thought to control the majority of plant-microbe interactions (Hacquard et al., 2017). If R proteins are stimulated in conjunction with PRRs, the plant can generate an amplified immune response called effector-triggered immunity (Jones and Dangl, 2006). While PTI can generate robust immunity independently of effector-triggered immunity, effector-triggered immunity requires PTI to mount meaningful resistance (Ngou et al., 2021; Yuan et al., 2021). Furthermore, PTI and effector-triggered immunity can have systemic, long-term effects on plant physiology by inducing systemic resistance (Mishina and Zeier, 2007; Pieterse et al., 2014). This causes distal, unexposed tissues to exhibit enhanced resistance to pathogen infection: a phenotype that can persist over many weeks and potentially into future generations (Luna et al., 2012).

Since MAMPs of numerous microbes are reactive with plant PRRs (Yu et al., 2019) and PTI is a central component in plant immune responses that affect microbial growth, MAMP-detecting PRRs may affect the structure of plant microbiomes. Experimental evidence from plant-microbe pairs supports the hypothesis. For example, single knockouts of many well-characterized MAMP-detecting PRRs in *A. thaliana* allow increased pathogen growth and/or increased disease severity (Zipfel et al., 2004; Wan et al., 2008; Nekrasov et al., 2009; Willmann et al., 2011; Wan et al., 2012; Ranf et al., 2015). Likewise, transforming plants with non-native PRRs can reduce pathogen growth and disease severity (Lacombe et al., 2010; Liu et al., 2021). In addition to suppressing pathogen growth, plant PRRs can also mediate the interaction between plants and beneficial microbes. For example, plant beneficial *Bacillus velezensis* requires PTI induced by the PRR EF-TU RECEPTOR (EFR), which detects a small fragment of bacterial elongation factor Tu, to efficiently colonize the *A. thaliana* root surface (Tzipilevich et al., 2021). Similarly, beneficial arbuscular mycorrhizal fungi requires stimulation of the PRR OsCERK1, which detects fungal chitin, to effectively colonize rice (Miyata et al., 2014).

Although MAMP-detecting PRRs clearly regulate the interactions of many plant-microbe pairs, how they sculpt the complex plant microbiome is less clear. In a complex community, the effect of the plant immune system on a given microbe may depend on the activity of other community members, including immunosuppression (Ma et al., 2021; Teixeira et al., 2021). Furthermore, plants respond in a distinct manner to particular types of MAMPs (Vetter et al., 2016). The baseline expression of MAMP-detecting PRRs and their downstream signaling pathways depends on the PRR in question, tissue type and developmental stage (Millet et al., 2010; Wan et al., 2012; Wyrsch et al., 2015; Rich-Griffin et al., 2020; Emonet et al., 2021; Verbon et al., 2023), thus the influence of PRRs on microbiome assembly may be specific to the present MAMPs and localized within an individual plant. Experiments evaluating the role of MAMP-detecting PRRs in *A. thaliana* microbiome assembly using synthetic microbial communities have yielded mixed results. Colaianni et al. (2021) found that root and shoot microbiomes were depleted in bacteria carrying immunogenic versions of the MAMP flagellin compared to microbial communities in surrounding agar. On the other hand, lab-based studies using complex synthetic communities rarely observe differences in microbiome structure in MAMP-detecting PRRs knockout lines compared to wild-type plants (Bodenhausen et al., 2014; Chen et al., 2020; Wippel et al., 2021; Wolinska et al., 2021). However, the synthetic communities used in these experiments were derived from microbes that closely associate with wild-type plants, potentially bypassing the filtering of environmental microbes mediated by PRRs. Other greenhouse-based experiments found some evidence of small effects of PRRs on plant microbiome structure using soil collected from the field (Wolinska et al., 2021; Fonseca et al., 2022). Fonseca et al. (2022) found that *A. thaliana fls2* mutant plants, which are unable to detect a MAMP derived from bacterial flagellin, assembled distinct rhizosphere communities compared to wild-type plants, but the rhizospheres of other PRR knockout lines (*efr* and *cerk1*, respectively) were indistinguishable from wild-type plants. Wolinska et al. (2021) found minor changes in endophytic (within tissue) bacterial root communities in the triple *fls2 efr cerk1* mutant compared to wild-type plants. Curiously, the triple mutant *bak1 bkk1 cerk1*, with dysfunctional coreceptors of these PRRs, had no apparent effect on bacterial community structure. Additionally, experiments using wild soil and a synthetic community derived from this soil identified only partially overlapping PRRs/PRR coreceptors as important factors in structuring microbial communities (Wolinska et al., 2021).

Several key questions remain concerning the role of PRRs in microbiome assembly. When exposed to the immense microbial diversity present in the field, do MAMP-detecting PRRs modulate microbiome structure? If so, is the effect specific to certain tissues or developmental stages? To address these questions, we grew wild-type *A. thaliana* Columbia-0 and four single knockout lines of MAMP-targeting PRRs, *fls2*, *efr*, *lore* and *lyk4*, in the field in southwest Michigan. Mutant plants were prevented or impaired from detecting various well-characterized MAMPs: epitopes from bacterial flagellin or elongation factor-Tu, certain bacterial lipids (medium-chain 3-hydroxy fatty acids and 3-hydroxyalkanoates), or fungal chitin (**Table 1**). Mutant lines were also previously shown to affect the growth of at least one microbe (Zipfel et al., 2004; Nekrasov et al., 2009; Wan et al., 2012; Ranf et al., 2015). Surface sterilized seeds were planted in flats filled with field soil in Fall 2017. Flats were placed into the field, where plants germinated, overwintered as rosettes, and bolted in the spring as is typical for local, wild *A. thaliana*. At four developmental stages (Vegetative, Flowering, Unripe Siliques, Ripe Siliques), all present plant tissues, including roots, rosettes, stems, cauline leaves, flowers, and siliques (seed pods), were harvested. We characterized the endophytic microbiome of each tissue because the microbial filtering effect is strongest in internal plant spaces. This experiment reveals a comprehensive picture of if, when, and where MAMP-detecting PRRs influence *A. thaliana* endosphere microbiome structure in the field.

**Table 1:** Pattern recognition receptors evaluated in this experiment.

Receptor Name	MAMP detected	Microbes affected	References
FLAGELLIN-SENSITIVE 2 (FLS2)	flg22 epitope from flagellin	bacteria	Zipfel et al., 2004
EF-TU RECEPTOR (EFR)	elf18 epitope from EF-Tu	bacteria	Zipfel et al., 2006; Nekrasov et al., 2009
LIPOOLIGOSACCHARIDE-SPECIFIC REDUCED ELICITATION (LORE)	medium-chain 3-hydroxy fatty acid metabolites/(R)-3-hydroxyalkanoate	bacteria	Ranf et al., 2015, Kutschera et al., 2019; Schellenberger et al., 2021
LYSM-CONTAINING RECEPTOR-LIKE KINASE 4 (LYK4)	chitin/?	fungi/bacteria	Wan et al., 2012

*A. thaliana* knockout lines of each of these receptors were planted in the field alongside wild-type plants. Bacterial and fungal microbiome composition was characterized across numerous tissues and developmental stages.

## 2 Materials and methods

### 2.1 Plant materials

Wild-type *A. thaliana* Columbia-0 (Col-0) and four PRR T-DNA insertion lines in the Col-0 background were used. The mutants *lore* (SAIL 857 E06) and *lyk4* (WISCDSLOX297300 01C) were obtained from the Arabidopsis Biological Resource Center. *fls2* (SALK 141277) was a gift from J. Greenberg and *efr* (SALK 044334) was a gift from S. Robatzek. All lines were previously confirmed to be null mutants and affect microbial growth *in planta* (Zipfel et al., 2004; Zipfel et al., 2006; Nekrasov et al., 2009; Wan et al., 2012; Ranf et al., 2015). Mutant lines were confirmed homozygous mutants by T-DNA amplification with the primers listed in **Table S1**.

### 2.2 Study site and planting

The field experiment occurred from October 2017 - May 2018 at the University of Chicago Warren Woods Ecological Field Station in southwest Michigan (41.83, -86.63). Seeds were surface sterilized with 50% bleach and stratified in sterile DI water for three days at 4°C. In late September, soil was collected from the field site and sifted with a 2 mm sieve to remove large debris. 36-cell flats were filled with the sifted soil and soaked with tap water. A plastic washer was placed in the center of each cell to mark target plants, and a single stratified seed was pipetted into the center of the washer. Plant genotypes were randomized across flats. Flats were placed in shallow holes in the field site and spaces between each cell loosely packed with soil. Drainage holes in the bottom of each cell allowed contact with the surrounding soil. Until the first true leaves emerged, flats were covered with plastic domes during rainstorms to prevent seeds from washing away, but left uncovered otherwise. Flats were initially watered daily with tap water for several weeks if required by weather conditions. In total, 35 flats with 1250 plants (250 replicates of each genotype) were planted. Plants germinated and overwintered as rosettes as is typical for local *A. thaliana* populations.

### 2.3 Sample collection and processing

Bulk soil from the four corners and the center of the experimental plot was sampled each day plants were harvested. Flame-sterilized tweezers were pressed 5 cm deep into the soil to extract a narrow core. Soil cores were placed into plastic storage tubes and immediately frozen at -80°C.

Plants were randomly selected for harvesting at several developmental stages described in **Table 2**. Plants were harvested in sets of five (one of each genotype) and immediately processed. Siliques of each plant were counted if present. Excess soil was removed by gently patting roots with a flame-sterilized metal spatula. Roots and aerial tissues were separated with a flame-sterilized razor blade and placed into a 50 mL conical tube with 25 mL of surfactant buffer (6.33 g NaH_2_PO_4_·H_2_O, 16.5 g Na_2_HPO_4_·7H_2_O, per 1 L, autoclaved then 200 µL Silwet L-77 added) (Lundberg et al., 2012). Epiphytes were removed based on protocols described in Lundberg et al. (2012) and Perisin (2016). Briefly, tubes were vortexted for 15 seconds, transferred to a fresh tube of buffer, and vortexed again for 15 seconds. Any remaining clumps of soil attached to plant tissues were removed by gently rinsing with additional surfactant buffer and/or using flame-sterilized tweezers. Aerial plant parts were separated using a flame-sterilize razor blade. The entire plant was retained with replicate parts combined into a single tube (e.g. all cauline leaves of an individual plant were combined into a single tube). Separated plant parts were transferred to fresh tubes of surfactant buffer; large plant parts in 50 mL conical tubes (25 mL surfactant buffer) and small plant parts in 1.7 mL Eppendorf tubes (1 mL surfactant buffer) and then sonicated using 30 second on/off cycles for a total of 5 minutes. Plant parts were transferred to storage tubes and immediately placed at -80°C until further processing. If samples were too large to fit into a single tube, they were spread across additional tubes.

**Table 2:** Tissues and developmental stages harvested.

Stage	Tissues present	Harvest dates	Number of whole plants harvested
Vegetative	root, rosette	March 9-10, 2018	40 (8 per genotype)
Flowering	root, rosette, stems, cauline leaves, flowers	April 21-23, 2018	40 (8 per genotype)
Unripe Siliques	root, rosette, stems, cauline leaves, flowers, immature siliques	May 6-15, 2018	90 (17-19 per genotype)
Ripe Siliques	root, rosette, stems, cauline leaves, flowers, immature siliques, mature siliques	May 15 - 23, 2018	40 (7-9 per genotype)

Whole plants were harvested in sets of five (one of each genotype). The number of samples derived from each plant is equivalent to the number of tissues present at the time of harvest (e.g. Vegetative plants each produced two samples: a root and a rosette sample). Developmental stages were defined as follows: Vegetative = no reproductive tissues, Flowering = flowers present without siliques, Unripe Siliques = siliques present but immature, Ripe Siliques = at least some are ripe siliques are present.

### 2.4 Spike-in sequences and design

Plasmids containing synthetic sequences that coamplify with ITS1 region of the fungal internal transcribed spacer region were acquired from Addgene (Tkacz et al., 2018), and synthetic sequences that coamplify the 799F - 1193R region of 16S were designed in-house (**Supplemental Methods, Section 1.4**). Plasmids were grown in *E. coli* and purified using QIAGEN MiniPrep kits. Known amounts of purified plasmid were added to the initial PCR reaction to allow absolute quantitation of microbial load across samples as described in Tkacz et al. (2018).

### 2.5 DNA extraction

Sample preparation and DNA extraction was performed as in Perisin (2016). Briefly, samples were lyophilized (LABCONCO FreeZone 4.5), weighed, and randomized across plates. Negative controls (TES: 10 mM Tris-Cl, 1 mM EDTA, 100 mM NaCl) and a synthetic control community composed of 10 microbes (ZymoBIOMICS Microbial Community Standard, D6300) were included in each extraction plate. Samples were homogenized by bead beating; 2-3 sterilized 2.3 mm silica beads were added to each tube, and samples were homogenized over two, 2.5 minute cycles at 1750 RPM in a homogenizer (2010 Geno/Grinder, SPEX). Samples that were not adequately homogenized were subjected to additional bead beating cycles using several 2.3 mm steel beads and/or manual grinding. Samples were suspended in TES at 0.05 mg sample per *µ*L, with a minimum volume of 250 *µ*L TES. Samples were homogenized once more at 1750 RPM for 2.5 minutes, and DNA was extracted using a double enzyme digest, chloroform/isopropanol precipitation (Perisin, 2016; **Supplemental Methods, Section 1.1**).

### 2.6 Mutant confirmation of field-grown plants

After DNA extraction, each plant sample was tested to confirm it matched the expected genotype using T-DNA insert amplification with the primers listed in **Table S1**. Only samples that were the expected genotype were included in the downstream analysis. Samples that appeared heterozygous for the T-DNA insertion (likely due to well-to-well cross-contamination) were excluded from the analysis.

### 2.7 Library preparation and sequencing

Amplicon libraries were generated using KAPA HotStart HiFi PCR kits (Roche), with custom Illumina primers with inline barcodes (**Tables S2–S4**). Briefly, in the first amplification round, the V5-V7 region of 16S ribosomal gene (Bodenhausen et al., 2013) or ITS1 (Horton et al., 2014) were amplified (**Tables S5, S6**). PCR products were purified with magnetic beads (**Supplemental Methods, Section 1.2**) and indexed with custom Illumina MiSeq indexing primers (**Tables S4, S7, S8**). PCR products were bead purified and quantified with Quant-iT PicoGreen dsDNA kits (Invitrogen) according to manufacturer’s instructions (3 *µ*L PCR product in 200 *µ*L total volume per sample). PCR products were pooled in equimolar amounts and concentrated (SpeedVac, ThermoFisher). Concentrated pools were size selected between 200-700 bp on a 1.5% agarose gel to remove primer dimers (BluePippin, Sage Science). Size-selected libraries were bead purified and library quality was assessed with a Bioanalyzer (High Sensitivity DNA, Agilent). Final libraries were sequenced on an Illumina MiSeq with a v3 2x 300 kit with ∼ 12% PhiX.

### 2.8 Data processing

Raw FASTQs were initially demultiplexed using the MiSeq onboard bcl2fastq2 software. Primer sequences were trimmed using cutadapt (paired 5’ primers, e=2.0, minimum length = 100 for both reads) (Martin, 2011). Each MiSeq run was processed separately until chimera removal, after which libraries of the same amplicon were pooled. For 16S libraries, truncation length and maximum expected error for DADA2 were determined using FIGARO on untrimmed reads (Sasada et al., 2020). ITS1 libraries were not trimmed. Reads were filtered, inferred, and merged using DADA2 (merging = minimum 40 bp overlap) to generate amplicon sequence variants (ASVs) (Callahan et al., 2016). Runs within amplicon type were combined and chimeras were removed with DADA2 (method = pooled). Sequences were classified to the genera level with Naive Bayes classifiers custom built with scikit-learn in QIIME2 (Bolyen et al., 2019). The 16S classifier was built using the SILVA-138 database (Quast et al., 2013), while the ITS1 classifier was built using the UNITE database (version 8) (Nilsson et al., 2018). Taxonomic trees were generated using MAFFT in QIIME2 (Bolyen et al., 2019).

Spike-in sequences were identified by BLAST alignment in QIIME2. Reads mapping to *E. coli* TOP10 16S sequence were removed from the analysis, as this strain was used to grow the plasmid carrying the spike sequence. After spike-in and *E. coli* read removal, plant-associated samples had a median sequencing depth of 11929 and 5089 reads for 16S and ITS1, respectively. Downstream analysis was performed in R (R Core Team, 2022) using the phyloseq package unless otherwise noted (McMurdie and Holmes, 2013).

### 2.9 Microbial load analysis and scaling for absolute quantitation

For overall load and absolute quantitation, only samples with spike reads representing between 20%-80% of the total read count were analyzed to ensure accurate quantitation (Tkacz et al., 2018). Experimental read counts were then scaled by the amount of spike-in sequences recovered using the following equation:


(1) $$Experimental_{scaled}=Experimental_{raw}\times \frac{Spike_{median}}{Spike_{sample}}$$

where *Experimental_raw_* are the number of experimental (non-spike) reads in the sample, *Spike_median_* is the median count of spike reads across the data set and *Spike_sample_* is the number of spike reads in the sample.

### 2.10 Quality filtering

For all community composition analyses using 16S and ITS1 data sets, samples with less than 500 reads were discarded. ASVs with less than 10 reads across the entire data set were also discarded. Senescent siliques were excluded from downstream analyses because their low biomass frequently resulted in poor DNA yields and representation in microbiome data set. In plant-associated samples, this quality filtering resulted in a median of 12404 or 5843 reads per sample in bacterial and fungal data sets, respectively.

### 2.11 *α*-diversity

Data was repeatedly rarefied to account for read depth variation (Cameron et al., 2021). Sampling depth was determined by analyzing rarefaction curves generated with the vegan package (Oksanen et al., 2022). Each data set was rarefied by sampling without replacement 100 times (sample depth: 16S = 1380, ITS1 = 751). Shannon Diversity (Shannon, 1948) and Pielou’s Evenness (Pielou, 1966) was calculated after each iteration for 16S and ITS1 data using the microbiome package (Lahti and Shetty, 2019). Faith’s Phylogentic Distance (Faith, 1992) corrected for species richness was calculated for the 16S data set using picante (Kembel et al., 2010). The mean *α*-diversity of each sample after 100 iterations was used in downstream analysis (Cameron et al., 2021).

Statistical analysis of *α*-diversity was performed using 3-way permutational ANOVA (Manly, 2007) using the following model where all terms interact:


(2) AlphaDiversity=Tissue∗Stage∗Genotype

### 2.12 Defining the core microbiome

We defined the core microbiome in three ways. Core A represents a global plant endophyte core, spanning all plant parts and developmental stages. ASVs with at least 0.5% relative abundance in four or more samples across the entire data set were retained. Core B was compiled from tissue and stage specific communities, since tissue type and developmental stage affects microbiome composition of *A. thaliana* at our field site (Beilsmith et al., 2021). Samples were subsetted by tissue type and stage score, and ASVs with ≥ 1% relative abundance in at least 20% of samples in at least one subset were retained. Finally, the Indicator Core was composed of ASVs enriched in the plant compared to the surrounding soil, determined by the indicspecies package (De Cáceres and Legendre, 2009).

### 2.13 *β*-diversity analysis

Several combinations of core filtering procedures, data transformations, and *β*-diversity indices were completed (summarized in **Table 3**). Two broad classes of transformations methods, rarefying and log-ratio transformations, were applied to the data sets.

**Table 3:** Data filtering, transformations, and diversity metrics used in β-diversity analysis.

Analytical variables for rarefied data	
Filtering levels	Core A, Core B, Indicator Core
Transformation methods	Repeat rarefy (compositional), Spike-in scaled repeat rarefy (absolute)
β-diversity metrics	Bray-Curtis, Jaccard, Weighted Unifrac*
Analytical variables for log-ratio transformed data	
Filtering levels	Core A, Core B, Indicator Core
Transformation methods	Robust center log-ratio (rCLR), Additive log-ratio (ALR) scaled by spike-in
β-diversity metrics	Euclidean (Aitchison)

*Performed on 16S data only.

#### 2.13.1 Rarefying

Sample depth varied over three orders of magnitude in these data sets. To mitigate spurious correlations generated by read depth variation, we repeat rarefied the ASV table 100 times (sample depth: 16S = 1380, ITS1 = 751) (Cameron et al., 2021). The mean ASV table of these iterations was used in downstream analyses. The rarefied table was filtered according to different core definitions described previously. For absolute abundance analyses, ASV counts were scaled using the ratio of spike-in reads to the total sample reads. Bray-Curtis Dissimilarity (Bray and Curtis, 1957) and Jaccard Index (Jaccard, 1912) were calculated for 16S and ITS1 data sets using vegan (Oksanen et al., 2022) and Weighted UniFrac (Lozupone et al., 2011) was calculated for 16S data only using QIIME2 (Bolyen et al., 2019).

#### 2.13.2 Log-ratio transformations

We also used methods appropriate for compositional data sets (Quinn et al., 2019) in parallel with the transformations described above. Transformations included the robust center log-ratio (rCLR) (Martino et al., 2019) and additive log-ratio (ALR) (Aitchison, 1986). For rCLR transformations, core microbiomes were scaled to the median read depth before transformation. For ALR calculations, core microbiomes ASV counts were scaled by the number of spike reads within the sample. The Euclidean distances between log-transformed communities were used for downstream cluster analyses (Quinn et al., 2019).

#### 2.13.3 Statistical analysis of *β*-diversity metrics

The factors influencing microbiome community structure in all resulting distance matrices were evaluated using PERMANOVA (Anderson, 2017) with the *adonis2* function in vegan (Oksanen et al., 2022), using the equation:


(3) Distance∼MiSeqRun:Plate+Tissue∗Stage∗Genotype

where the DNA extraction/PCR plate nested in MiSeq run is considered a random effect, and tissue, stage, and genotype are fixed effects in a three-way interaction.

### 2.14 Differential abundance

Differential abundance analysis across the overall data set was performed using ANCOM-BC2 (Lin and Peddada, 2020). Untransformed (raw counts) core microbiomes were analyzed in ANCOM-BC2. The effect of genotype was tested with the model:


(4) Abundance∼Tissue+Stage+Genotype

We also manually tested the interactions between genotype, stage, and tissue. To accomplish this, the data were subsetted by tissue, stage, and tissue by stage, and reanalyzed for a genotype effect.

Targeted evaluation of differential abundance between *lore* and wild-type lineages at Ripe Siliques developmental stage was performed using ANCOM-BC2 (Lin and Peddada, 2020) and DESeq2 (Love et al., 2014). DESeq2 was performed by using filtered data sets (Core B) using a zero-tolerant geometric mean (zeros ignored) to estimate size factors. ANCOM-BC2 analysis was performed as described above, except the model was adjusted to:


(5) Abundance∼Tissue+Genotype

### 2.15 Within genotype microbiome dispersion

To test if microbiome community structure was equally variable within different genotypes, the genotype group dispersion was calculated using PERMDISP2 (Anderson et al., 2006), implemented using the *betadisper* and *permutest* functions in vegan (Oksanen et al., 2022). This analysis was applied to distance matrices generated by all microbiome cores, transformation methods, and *β*-diversity indices generated in previous sections. Additionally, dispersion was evaluated in a minimally filtered data set to capture variability derived from rare community members. Since PERMDISP2 can only be applied to models with a single factor, we evaluated the dispersion of different genotypes across all tissues and stages, as well as genotype within stage, within tissue, and within tissue by stage subsets to test for an interaction between genotype and other fixed factors.

### 2.16 Microbiome variation within individual plants

We tested if microbiomes derived from different tissues of the same individual plant were more similar to one another in PRR knockout lineages compared to wild type plants. Three different tissue subsets were analyzed: 1) all tissue types (roots, rosettes, stems, cauline leaves, flowers, and siliques) to cover all within-plant tissue variation but limited to the final developmental stages, 2) roots and rosettes only to assess all developmental stages, and 3) all aerial tissues except siliques, because above-ground selective pressures are highly distinct from below-ground pressures. Only individual plants with all relevant tissue types present in the data set were considered in each analysis.

After selecting appropriate samples, distance matrices were generated using Bray-Curtis dissimilarity. The *betadisper* function was used to ordinate these data and calculate the distance between the median community of an individual plant and each of its associated tissues (Oksanen et al., 2022). The mean of these distances used to quantify tissue similarity within individuals. A permutational ANOVA was used to determine if stage or genotype affected within-individual community similarity with the following equation:


(6) MeanDistanceToMedian∼Stage∗Genotype

### 2.17 Early fitness analysis

Two fitness proxies for vegetative biomass and seed output (Violle et al., 2007) were assessed: rosette dry weight (vegetative biomass) and silique count (seed production). Rosette dry weight and siliques counts were assessed during sample processing as described in Section 2.3. Plants were harvested before all siliques emerged, thus this assay measured only early fitness. As expected, rosette dry weight and total silique count is correlated when plant age is considered, except in the final week of the experiment when sample size was small (Pearson’s correlation, *p <* 0.05, **Figure S1**).

## 3 Results

### 3.1 A single PRR knockout does not affect endophytic microbiome *α*-diversity

**Figure 1:**
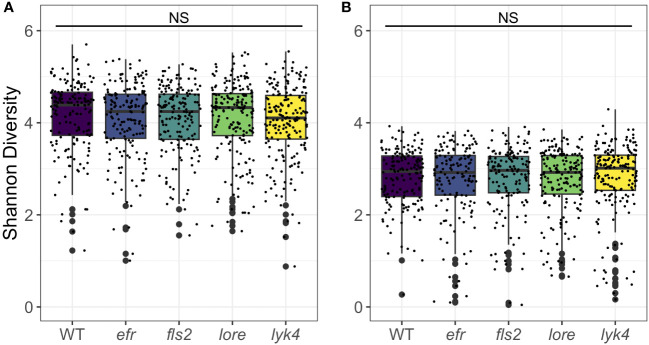
PRR knockout has no significant effect on Shannon diversity of bacterial or fungal microbiomes. PRR knockouts *efr, fls2, lore*, and *lyk4* do not have significantly different Shannon diversity than wild-type plants in **(A)** bacterial or **(B)** fungal communities, either as a main effect (shown here, permutational ANOVA, *p >* 0.05) or in interactions with tissue and stage (permutational ANOVA, *p >* 0.05; **Tables S9** and **S10**). Bacteria, genotype main effects *n*=166-200; genotype by tissue by stage subsets *n*=3-18. Fungi, genotype main effects: *n*=143-183; genotype by tissue by stage subsets: *n*=2-17. Statistical difference between genotypes (main effect) in a global permutational ANOVA: NS = not significant.

Endophytic microbiomes are composed of only a subset of environmental microbes (Bulgarelli et al., 2012; Lundberg et al., 2012; Wippel et al., 2021). As front-line mediators of plant-microbe interactions, MAMP-detecting PRRs may contribute to this effect. We tested if the endosphere microbiomes of PRR mutant plants had increased *α*-diversity, which could indicate less plant selectivity. We calculated Shannon Diversity (Shannon, 1948) on rarefied 16S and ITS1 data sets and Faith’s Phylogenetic Distance scaled for species richness (Faith, 1992) on the rarefied 16S data set. To account for data loss from rarefying, rarefying and subsequent diversity calculations were repeated 100 times and the mean diversity score was used in statistical analyses (Cameron et al., 2021). There was no difference in Shannon diversity between PRR knockouts and wild-type plants in bacterial or fungal microbiomes (three-way permutational ANOVA, *p >* 0.05; **Figure 1**; **Tables S9** and **S10**) or the Faith’s phylogenetic distance of bacterial communities (three-way permutational ANOVA, *p >* 0.05; **Figure S2**; **Tables S9** and **S10**). Tissue type, developmental stage, and the interaction between these factors affected *α*-diversity (*p <* 0.05). We also considered the possibility that MAMP-detecting PRRs preferentially exclude high-growth, pathogenic microbes. If PRR knockout allows previously excluded pathogens to infiltrate and then dominate the microbiome, community evenness - the distribution of abundances of the species in the community - may be affected. However, we did not find any support for this supposition; Pielou’s evenness in bacterial and fungal microbiomes is indistinguishable between PRR knockouts and wild-type plants (three-way permutational ANOVA, *p >* 0.05; **Figure S3**; **Tables S9** and **S10**).

### 3.2 A single PRR knockout has a small effect on fungal, but not bacterial, endosphere microbiome composition

**Figure 2:**
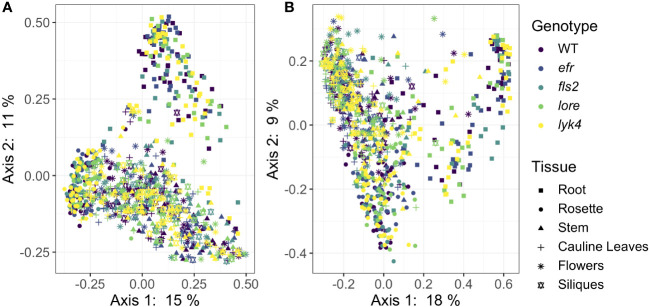
PRR knockout has subtle effects on Bray-Curtis *β*-diversity of endophytic fungal microbiomes, but not bacterial microbiomes. Principle Coordinate Analysis (PCoA) of Bray-Curtis distances between bacterial **(A)** or fungal **(B)** microbiomes. **(A)** PRR mutations, denoted by color, do not explain community variation in Bray-Curtis distances in endophytic bacterial communities as a main effect or as an interaction with tissue and/or stage (PERMANOVA, *p >* 0.05). **(B)** PRR genotype had subtle effects on Bray-Curtis distance in fungal communities, but this effect is not obvious on primary PCoA axes (PERMANOVA, *R* ^2^ = 0.005, *p <* 0.05). In accordance with previous work at this field site (Beilsmith et al., 2021), microbial communities segregate by tissue type (represented by shape) on primary PCoA axes. Tissue type had a substantial effect on community composition (PERMANOVA, bacteria: *R* ^2^ = 0.183, *p <* 0.05; fungi *R* ^2^ = 0.121, *p <* 0.05). Bacteria: *n*=3-18 for each genotype by tissue by stage subset, with *n*=888 total samples. Fungi: *n*=2-17 for each genotype by tissue by stage subset, with *n*=816 total samples.

We then asked if PRRs affected the *β*-diversity of endosphere microbiome composition. We evaluated the significance of genotype, tissue type, and developmental stage on core endosphere microbiome structure using several different approaches targeting different features of *β*-diversity (**Table 3**). PRR knockout had a small effect on endophytic fungal communities and interacted with developmental stage (**Figure 2**, Bray-Curtis, *R* ^2^ = 0.0044, *p <* 0.05, **Table S13**), although this effect was detected in only some *β*-diversity metrics (**Tables S13, S14**). Post-hoc analyses revealed that genotype affected endosphere fungal communities in the Unripe Siliques and Ripe Siliques stages (PERMANOVA, *p <* 0.05 and *p <* 0.05, respectively). Pairwise comparisons showed that *lore* knockouts had statistically different fungal communities than wild-type plants in the Ripe Siliques stage (Bray-Curtis, WT vs. *lore* pairwise PERMANOVA within the Ripe Siliques developmental stage, *R* ^2^ = 0.015, *p <* 0.05). This was unexpected because *LORE* detects bacterial medium-chain 3-hydroxy fatty acids and 3-hydroxyalkanoates (Kutschera et al., 2019; Schellenberger et al., 2021), but has no documented effect on fungi. However, it is possible that *LORE* also detects fungal lipids/MAMPs - other PRRs detect multiple elicitors and affect plant interactions with both fungi and bacteria (Willmann et al., 2011; Wan et al., 2012). Alternatively, the bacterial microbiome has been shown to strongly influence fungal microbiome structure (Durán et al., 2018); if *LORE* transiently affected the bacterial community, this may have had cascading effects on the fungal microbiome. Other notable but statistically insignificant differences in fungal community composition between wild-type and PRR knockouts include *lore* in the Unripe Siliques stage and *lyk4*, a chitin-responsive PRR, in the Ripe Siliques stage (Bray-Curtis, pairwise PERMANOVA: *R* ^2^ = 0.0081, *p* = 0.069; *R* ^2^ = 0.041, *p* = 0.067, respectively). Finally, genotype effects were only detectable on the ASV taxonomic level (data not shown). In contrast, genotype had no effect on bacterial *β*-diversity across all core communities, transformation methods and diversity metrics (**Figure 2**, PERMANOVA, *R* ^2^ = 0.005*, p >* 0.05, **Supplemental Tables S11, S12**). Additionally, genotype had no effect on bacterial community composition at higher taxonomic levels (data not shown). Considering all of the *β*-diversity analyses together, we found that PRRs have little effect on endosphere microbiome *β*-diversity as effects were limited to a single genotype and developmental stage within the fungal microbiome.

We next attempted to uncover which fungal ASVs drove the shift in *β*-diversity of *lore* mutants in the Ripe Siliques stage by testing for differentially abundant ASVs using ANCOM-BC2 (Lin and Peddada, 2020) and DESeq2 (Love et al., 2014). Neither analysis detected any fungal ASVs with statistically significant changes in abundance between wild-type plants and *lore* mutants (ANCOM-BC2, *p >* 0.05; DESeq2, *p >* 0.05), which was unsurprising given the small Bray-Curtis effect size of this comparison (Nearing et al., 2022; effect size determined by PERMANOVA: *R* ^2^ = 0.015). We also evaluated if any bacterial or fungal ASVs were differentially abundant between wild-type and PRR knockout lineages across the entire data set using ANCOM-BC2 (Lin and Peddada, 2020). No bacteria or fungi were differentially abundant when genotype was considered as a main effect, nor when genotype was tested within tissue, developmental stage, or tissue by stage subsets (ANCOM-BC2, *p >* 0.05).

### 3.3 Single PRR knockouts and wild-type plants show no difference in microbiome variability

**Figure 3:**
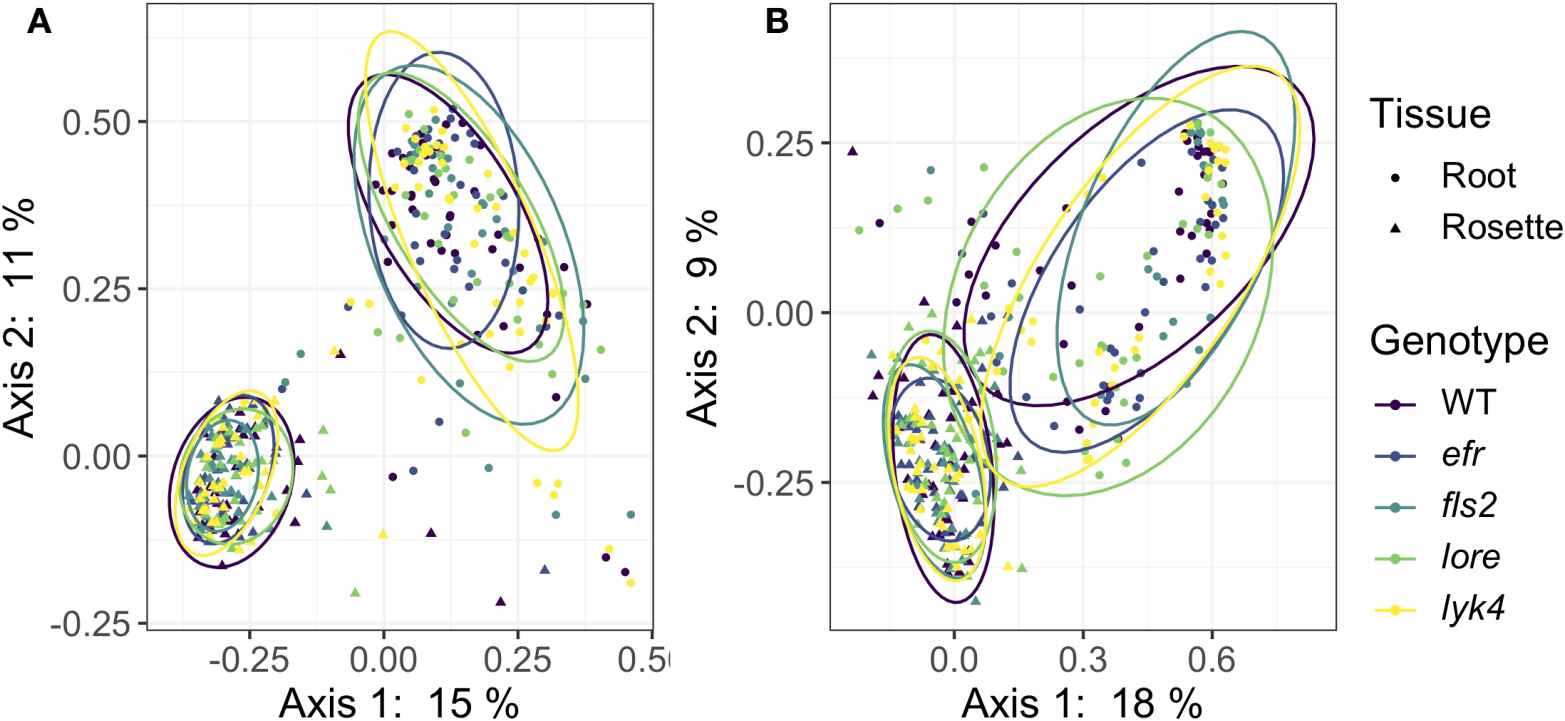
PRR mutant microbiomes are not more variable than wild-type microbiomes. PCoA of Bray-Curtis distance of **(A)** bacterial and **(B)** fungal communities. Only roots and rosettes are shown. To visualize group dispersions, ellipses encircle the 85% confidence interval t-distribution of samples in a genotype group, and are colored according to genotype. Genotype has no statistical effect on within-group microbiome variation (PERMDISP2, *p >* 0.05). Further, there is no effect of genotype on microbiome variability within tissue, stage, or tissue by stage subsets (PERMDISP2, all subsets, *p >* 0.05). Bacteria: *n*=3-18 for each genotype by tissue by stage subset, with *n*=888 total samples. Fungi: *n*=2-17 for each genotype by tissue by stage subset, with *n*=816 total samples.

Plant control of the microbiome can manifest in numerous ways. Host selection is a deterministic force governing microbiome assembly (Bulgarelli et al., 2012; Lundberg et al., 2012; Horton et al., 2014; Tkacz et al., 2020; Wippel et al., 2021; Brachi et al., 2022). If the host plant is unable to effectively select microbes, variability (i.e. dispersion) in microbiome structure between individuals could increase as stochastic processes, such as microbial dispersal and drift, become more important in community assembly (Arnault et al., 2022). Thus, if PRRs contribute to host control of the microbiome, within-genotype microbiome variability in PRR mutants may be increased compared to variability between wild-type plants. To test this, we compared *β*-diversity dispersion of each genotype using the PERMDISP2 procedure (*betadisper* function in vegan, Oksanen et al., 2022). Neither bacterial nor fungal communities were more variable within PRR mutants than within wild-type plants, even if the effects of tissue and stage were controlled (**Figure 3**, PERMDISP2 analysis of multivariate homogeneity of group dispersions, *p >* 0.05, **Table S15**). This indicates that single PRRs are not required for deterministic selection of environmental microbes.

### 3.4 The degree of tissue specificity in endophytic microbiome structure is not affected by the loss of individual PRRs, but changes over time

PRRs and plant immunity may help maintain the distinct microbial communities found in each tissue via two mechanisms. First, the expression patterns of MAMP-detecting PRRs and the regulation of downstream immune signaling pathways is cell-type specific (Millet et al., 2010; Wan et al., 2012; Beck et al., 2014; Vetter et al., 2016; Rich-Griffin et al., 2020; Emonet et al., 2021; Verbon et al., 2023). Second, PTI may impede the systemic spread of microbes (Yadeta and Thomma, 2013; Beck et al., 2014; de Lamo et al., 2021). Interestingly, there is evidence in humans that within-individual site-specificity of microbiomes declines in disease; critically ill patients exhibit reduced body-site specificity (Rogers et al., 2016). We tested if PRRs help maintain tissue specificity within individual plants by calculating Bray-Curtis distances between each tissue within each plant, and then calculating the mean distance from the community of each tissue to the median community of the individual plant. A two-way permutational ANOVA with genotype and development stage as fixed effects was used to test statistical significance.

**Figure 4:**
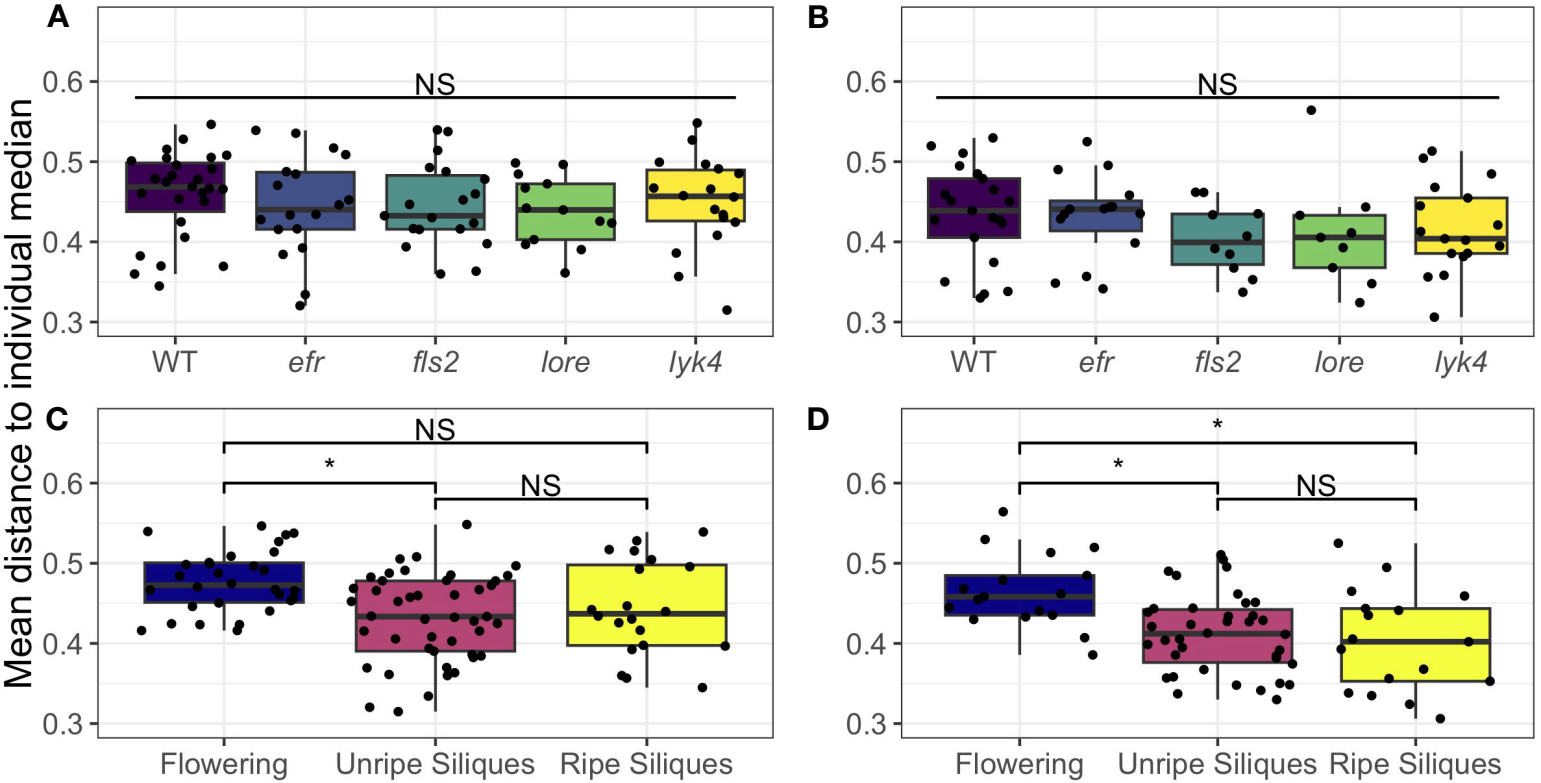
Microbial communities of aerial tissues within individuals do not show different degrees of site-specificity between PRR mutants wild-type plants, but generally become more similar as plants age. The mean Bray-Curtis distance from the microbial communities of the rosette, stems, cauline leaves, and flowers to the individual median community was calculated to measure within-individual tissue specificity. Within-individual tissue specificity does not vary by genotype in **(A)** bacterial (permutational ANOVA, *p >* 0.05) or **(B)** fungal communities (permutational ANOVA, *p >* 0.05). This is true for main effects (shown above; bacteria, n= 13-27; fungi, *n*=9-21) or interactions with stage (**Table S16**). Significant differences between genotypes according to global permutational ANOVA: * = *p* < 0.05, NS = not significant. However, developmental stage affects within individual site-specificity in both **(C)** bacterial communities and **(D)** fungal communities (permutational ANOVA main effect, bacteria: *p <* 0.05, *n*=20-45; fungi: *p <* 0.05, *n*=17-38). In **(C)** bacterial communities, the mean distance of each tissue’s microbiome to the plant median community decreased between the Flowering (no siliques present) and Immature Siliques stages, thus tissues became more similar (pairwise permutational ANOVA post-hoc with Benjamini-Hochberg correction, *p <* 0.05). However, this trend did not hold in the Mature Siliques stage (pairwise permutational ANOVA post-hoc, *p >* 0.05 after Benjamini-Hochberg correction). In fungal communities **(D)**, tissue specificity was significantly higher in the Flowering stage than both the Immature Siliques and Mature Siliques stages (permutational pairwise ANOVA post-hoc with Benjamini-Hochberg correction *p <* 0.05). Significant differences between developmental stages according to pairwise permutational ANOVA with B-H correction: * = *p* < 0.05, NS = not significant.

We found that wild-type plants and PRR knockout lines had the same degree of tissue specificity of both fungal and bacterial microbiomes within individual plants (**Figure 4**; **Table S16**). Interestingly, we found that both fungal and bacterial microbiomes of aerial tissues, excluding siliques, generally became more similar within individuals as plants matured (**Figure 4**, permutational ANOVA *p <* 0.05, with pairwise permutational ANOVA post-hoc tests; **Table S16**). Thus, although the microbiomes of most aerial tissues become more similar within individuals over time, we found no evidence that single PRRs play a direct role in regulating microbiome tissue specificity in *A. thaliana*.

### 3.5 No evidence of increased microbial load or reduced fitness in single PRR knockouts

**Figure 5:**
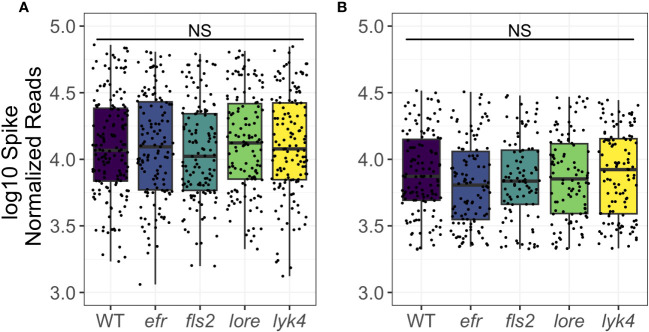
PRR knockout does not affect bacterial or fungal load. Microbial load was calculated by adjusting microbial read counts to synthetic spike-in read counts. Wild type and PRR knockout plants do not have significantly different microbial loads of bacteria **(A)** or fungi **(B)** (ANOVA, *p >* 0.05). Shown are main effects, but no interaction with tissue or stage was detected (**Table S17**). Bacteria, genotype main effects *n*=129-155; genotype by tissue by stage subsets *n*=2-15. Fungi, genotype main effects: *n*=93-123; genotype by tissue by stage subsets: *n*=2-17. Significant differences between genotypes according to global ANOVA: * = *p* < 0.05, NS = not significant.

A single PRR knockout can increase plant microbial load in single-microbe infections (Zipfel et al., 2004; Wan et al., 2008; Nekrasov et al., 2009; Willmann et al., 2011; Wan et al., 2012; Ranf et al., 2015). It is critical that plants regulate the total microbial load, as high microbial loads are associated with reduced fitness in the field (Traw et al., 2007). We thus asked if PRRs regulate overall microbial load and if PRR mutants have altered early fitness indicators. To estimate microbial load, a known amount of synthetic spike-in DNA that co-amplified with 16S or ITS1 was added to the initial PCR reaction. This allowed us to estimate total microbial load by scaling total read counts by the number of spike sequences (Tkacz et al., 2018). We detected no change in either bacterial or fungal load in PRR knockouts compared to wild-type plants either as a main effect or in interactions with tissue and stage (bacterial load: ANOVA, *p >* 0.05, **Figure 5A**; fungal load: ANOVA, *p >* 0.05, **Figure 5B**; **Table S17**). We also tested if the loss of a MAMP-detecting PRR affected plant fitness, which may be expected if PRR loss leads to increased susceptibility to pathogens and/or microbiome dysbiosis. However, we failed to find evidence that loss of MAMP-detecting PRRs impacted early silique counts (**Figure S4A**: Kruskal-Wallis, *p >* 0.05) or rosette dry weight (**Figure S4B**: Kruskal-Wallis, *p >* 0.05), although a small sample size limited our power to detect fitness differences. Thus, we found no evidence that individual MAMP-detecting PRRs control total microbial load in the field, nor that PRRs have large effects on early plant fitness.

## 4 Discussion

Complex microbial communities assemble on and within plant tissues, influencing plant phenotype. A key aim of many research programs is to effectively engineer these plant-associated microbiomes to achieve agricultural objectives, such as increased yield or resilience to abiotic and biotic stresses. Elucidation of microbial community assembly rules has the potential to improve the efficiency and reproducibility of these efforts.

Plant-associated microbiomes are comprised of only a subset of the microbes present in the environment, suggesting that plants filter and/or select their associated microbes. Plant immunity, which includes pattern recognition receptors that detect microbial MAMPs, is thought to sculpt plant microbiomes (Kniskern et al., 2007; Traw et al., 2007; Carvalhais et al., 2015; Lebeis et al., 2015; Hacquard et al., 2017; Colaianni et al., 2021; Kudjordjie et al., 2021; Parys et al., 2021; Fonseca et al., 2022). Indeed, in plant interactions with single microbes, individual MAMP-detecting PRRs can affect the colonization and *in planta* growth of particular bacteria or fungi (Zipfel et al., 2004; Nekrasov et al., 2009; Vetter et al., 2012; Wan et al., 2012; Ranf et al., 2015; Colaianni et al., 2021; Parys et al., 2021). However, the impact of PRRs on the assembly of complex endophytic microbial communities in the field is unknown. We characterized both bacterial and fungal endophytic microbiomes of wild-type *A. thaliana* and MAMP-detecting PRR knockout lines grown in the field, across several developmental stages and plant parts. This unprecedented scope allowed us to determine if, when, and where individual MAMP-detecting PRRs shape the endophytic microbiome in the field.

We found little evidence that individual MAMP-detecting PRRs impact endophytic microbiome structure despite measuring several *α*-diversity and *β*-diversity metrics, the variability in microbiome composition, the degree of tissue differentiation within individual plants, and the estimated total microbial load. We also failed to find an impact of PRRs on early plant fitness indicators. Indeed, we found no effect of PRR knockouts on the composition of bacterial communities and, for fungal communities, only Bray-Curtis and Jaccard diversity were altered in PRR knockout lineages (both *R* ^2^ = 0.005, *p* = 0.03; **Table S11**). Post-hoc analyses revealed that *lore* mutants hosted slightly modified fungal communities compared to wild-type plants, potentially revealing a role for *LORE* in plant-fungal interactions. Field data suggests that fungal communities can be affected by host factors that do not impact the bacteria community (Horton et al., 2014; Bergelson et al., 2019; Brachi et al., 2022) and that fungal communities are more sensitive to host effects than bacterial communities (Bergelson et al., 2019). A restricted impact of PRRs on fungi is furthermore consistent with analyses of co-occurrence networks suggesting that most microbe-microbe effects in wild *A. thaliana* occur within kingdom (Agler et al., 2016; Bergelson et al., 2019; Brachi et al., 2022).

There are several possible explanations for the general lack of effect of PRRs on microbiome community structure. First, redundancy in the plant immune system may maintain robust plant immune responses despite the loss of a single PRR. Members of microbial consortia produce diverse MAMPs that induce PTI to varying degrees (Garrido-Oter et al., 2018; Colaianni et al., 2021; Parys et al., 2021). Although loss of an individual PRR allows increased microbial proliferation in some single-microbe infections (Zipfel et al., 2004; Wan et al., 2008; Nekrasov et al., 2009; Willmann et al., 2011; Wan et al., 2012; Ranf et al., 2015), the presence of other microbes eliciting PTI via other intact PRRs may compensate for this effect. In nature, the plant responds to a complex input of MAMPs, DAMPs, effectors and other signals. Compellingly, recent work demonstrated that MAMP signaling must coincide with cellular damage to generate substantial PTI (Zhou et al., 2020). Thus, depending on the combination of signals produced by the microbiome, commensals may largely avoid activating PTI.

There is also considerable evidence that many plant-associated microbes have the ability to suppress plant immune responses and that this facilitates the colonization of PTI-triggering microbes (Teixeira et al., 2019; Ma et al., 2021). Three independent surveys (Yu et al., 2019; Ma et al., 2021; Teixeira et al., 2021) found that 31%-42% of plant-associated bacteria suppress PTI. This trait spans broad taxonomic categories and, importantly, the impact of suppressive strains dominates that of nonsuppressive strains in mixed bacterial communities (Teixeira et al., 2019; Ma et al., 2021). Considering the frequency, taxonomic diversity, and dominance of this trait, immunosuppressive microbes almost certainly affected community assembly in our natural microbiomes. If the anti-microbial response generated by stimulating PRRs is dampened by the endophytic microbiome, loss of a PRR would have little effect on subsequent microbiome assembly, as observed in our experiment. In this case, other aspects of plant-microbe associations such as plant structural components, bacterial metabolism, and microbe-microbe interactions (Horton et al., 2014; Bai et al., 2015; Levy et al., 2018; Salas-González et al., 2021; Velásquez et al., 2022) would have relatively more influence on commensal microbiome structure.

This study provokes two related questions. First, if PRRs are effectively redundant, why does selection maintain multiple PRRs? Second, if pattern triggered immunity is broadly suppressed, why maintain PRRs at all? One hypothesis (Hacquard et al., 2017) is that rather than filtering microbes from the environment, PRRs help regulate the total microbial load of the commensal microbiome to prevent damaging overgrowth. We found no evidence that single MAMP-detecting PRR knockouts supported higher microbial loads in the field (**Figure 5** and **Table S17**). Related experiments using PRR and PRR coreceptor multi-mutants report conflicting impacts of these genes on microbial load, both within and between experiments (Xin et al., 2016; Wolinska et al., 2021). This inconsistency suggests that PRRs regulate the microbial load of some communities, but that this is not a general effect.

An alternative hypothesis is that individual MAMP-detecting PRRs are maintained by selection from virulent pathogens or mutualists, rather than from interactions with commensals. Aggressive pathogen growth is typically accompanied by other signals such as DAMPs and effectors, which may allow the plant to overcome any background suppression of PTI. These pathogens are often controlled by powerful effector-triggered immunity which requires sustained PTI signaling to adequately function (Ngou et al., 2021; Yuan et al., 2021). Thus, pathogens may exert selective pressure on the specific subset of PRRs they activate. Since different pathogens activate overlapping PRRs (Zipfel et al., 2006; Wan et al., 2012; Ranf et al., 2015; Colaianni et al., 2021; Parys et al., 2021), each receptor could be maintained through interactions with numerous pathogens, even if encounters with a particular pathogen species are infrequent. Another possibility is that mutualisms exert selective pressure on specific PRRs. For example, orthologs of the PRR *CERK1* are required for both defense against pathogenic fungi and establishing mutualisms with arbuscular mycorrhizal fungi (AMF) in several distantly related plant species (Miyata et al., 2014; Bozsoki et al., 2017; Feng et al., 2019; Gibelin-Viala et al., 2019; Zhang et al., 2019). In these scenarios, the effect of PRRs on microbiome composition would only be detectable in the presence of virulent pathogens or important mutualists. Thus, results between different microbiomes could be inconsistent, as has been observed in this study and others (Bodenhausen et al., 2014; Chen et al., 2020; Wippel et al., 2021; Wolinska et al., 2021; Fonseca et al., 2022). Finally, specific PRRs may be maintained due to pleiotropic effects. For example, *CERK1* appears to have a conserved role in promoting lateral root formation in numerous plants, including *A. thaliana*, independent of accommodating an AMF mutualism (Chiu et al., 2022). Other PRRs may have developed additional functions, especially since MAMP-detecting PRRs are already integrated into growth-defense signaling pathways (Huot et al., 2014).

Finally, other biological and technical factors could explain why we detected few effects of PRRs on microbiome structure in the field. Myriad environmental conditions including temperature, humidity, soil salinity, phosphorus availability, and drought are known to modulate the strength of plant immunity and affect microbiome composition (Cheng et al., 2013; Castrillo et al., 2017; Naylor et al., 2017; Santos-Medellín et al., 2017; Berens et al., 2019; Chen et al., 2020). Although the field conditions in our experiment were representative of Midwestern USA, an area in which *A. thaliana* is common (Platt et al., 2010; Exposito-Alonso et al., 2018; Shirsekar et al., 2021), it is possible that PRR signaling was rendered unimportant by environmental conditions. Nevertheless, two lines of evidence suggest that our results may be generalizable. First, we characterized endophytic microbiomes over several time points, which would mitigate the chance of mischaracterizing the effects of plant immunity due to short-term environmental fluctuations. Second, while genome-wide association analyses on field-grown *A. thaliana* across years and locations occasionally identify known PRRs as candidate genomic features that affect microbiome composition, these effects are limited to one or two specific microbes rather than overall community composition and are often ephemeral (Horton et al., 2014; Brachi et al., 2022; Roux et al., 2023). We also cannot rule out the possibility that our lack of signal is a result of technical limitations. For example, the immunogenicity of flagellin is broadly linked to taxonomy (Colaianni et al., 2021; Parys et al., 2021), but the substantial within-genera and within-species variation of flagellin epitopes, and their capacity to trigger PTI, is unlikely to be resolved by 16S marker-gene sequencing (Vetter et al., 2016; Colaianni et al., 2021; Parys et al., 2021). In addition, PRRs may impact microbial subcommunities within tissues due to their localized, cell-type specific responses (Millet et al., 2010; Rich-Griffin et al., 2020; Emonet et al., 2021; Verbon et al., 2023); assessing microbiome structure of whole plant parts, as we did in this experiment, may mask these effects. Finally, we did not test every MAMP-detecting PRR identified in *A. thaliana*. However, even if other PRRs actively shape the commensal microbiome, why selection maintains the PRRs assessed in this experiment remains an important question.

In conclusion, we demonstrate that individual PRRs have little effect on the overall endophytic bacterial and fungal microbiome in *A. thaliana* in the field, as measured at the level of 16S and ITS1 characterization. Although initially surprising, these results offer valuable insight into the function of MAMP-detecting PRRs and help target the search for plant genetic factors that affect microbiome assembly in the field. Further investigation of hypotheses concerning the role of plant immunity in structuring microbiomes will improve our understanding of plant-microbe interactions, leading to a deeper understanding of these important ecological processes and more effective engineering of the plant microbiome.

## Data availability statement

The data presented in this study are deposited in the NCBI SRA repository, accession number PRJNA1015384. Scripts and commands used in data processing and analysis are available at https://github.com/carolineoj/MAMPR_microbiome.

## Author contributions

CO-J: Conceptualization, Formal Analysis, Investigation, Writing – original draft, Writing – review & editing. FH: Investigation, Writing – review & editing. JB: Conceptualization, Funding acquisition, Supervision, Writing – review & editing.
