# Supplementary material for: Microbe-associated molecular pattern recognition receptors have little effect on endophytic Arabidopsis thaliana microbiome assembly in the field
Source: Front Plant Sci. 2023 Nov 8;14:1276472. doi: 10.3389/fpls.2023.1276472 (PMC10663345; doi:10.3389/fpls.2023.1276472)
Supplement: Supplementary file 1 [file DataSheet_1.pdf]

Supplementary Material

1 SUPPLEMENTAL METHODS

1.1 DNA extraction

Lyophilized, homogenized plant tissues were suspended in TES at 0.05 mg sample per μL, with a minimum volume of 250 μL TES (10 mM Tris-Cl, 1mM EDTA, 100mM NaCl). 2-3 2.33 mm sterilized, silica beads were added to each tube, and tubes were homogenized for 2 minutes at 1750 RPM in a Spex GenoGrinder2000. 100 μL lysozyme mix (EpiCenter ReadyLyse lysozyme diluted to 125 U/μL in TES) was added to each well, gently vortexted for 10 seconds, and incubated at room temperature for 30 minutes. 250 μL Proteinase-K mix (0.5 mg/mL Proteinase-K, 1% SDS, to volume in TES) was added to each well, vortexed for 10 seconds to mix, and incubated at 55°C for 4 hours. 500 μL 24:1 chloroform:isoamyl was mixed into each well, and plates were centrifuged for 15 minutes at 6600 *x g* at 4°C. The top 350 μL of each well was added to plates with 500 μL cold 100% isopropanol, mixed, and then incubated at -20°C for 1 hour. Plates were centrifuged for 15 minutes (4°C, 6600 *x g*), isopropanol removed, and DNA pellets were washed with 400 μL 70% ethanol. Ethanol was removed and samples were dried in a biological safety cabinet using a 96-well blower to remove any remaining ethanol. DNA was then resuspended in 100 μL TE (10 mM Tris-Cl, 1mM EDTA) by vortexting for 2 minutes. To remove excess carbohydrates, plates were incubated on ice for 5 minutes and then centrifuged for 12 minutes (4°C, 6600 *x g*). 75 μL of the supernatant containing the DNA was removed and placed into fresh plates for further analysis.

1.2 Bead clean up

Bead clean ups were performed with in-house Solid Phase Reversible Immobilization (SPRI) beads (Rohland and Reich, 2012), which contain:
0.1% SpeedBead Carboxylate-Modified Magnetic Particles (Hydrophobic) (rinsed in TE buffer)
18% PEG-8000 (w/v)
1M NaCl
10mM Tris-HCl pH 8.0
1 mM EDTA, pH 8.0.

Clean up was performed as described in (Rohland and Reich, 2012) with the following parameters: beads were added at 1:1 ratio of SPRI solution to PCR volume, two rinses 80% EtOH were used, and the final genomic material was eluted in molecular-grade, sterile water.

1.3 Primers

1.3.1 Plant genotype confirmation primers.

Genotype	Primer	Sequence
efr	Forward	CTGTGGTGGTTAGGGATTCG
	Reverse	GATGGGTTACCATCACTGGC
	Insert	**LBb1.3:** ATTTTGCCGATTTCGGAAC
fls2	Forward	AGGGCTTCTTACAAACCTTCG
	Reverse	CGTTGATGTTTTTGAACACCC
	Insert	**LBb1.3:** ATTTTGCCGATTTCGGAAC
lore	Forward	CATTTTCATCCATCGATGGAC
	Reverse	TTCCCTTTCACAACAATCCTG
	Insert	**SAIL-LB1short:** TGGATAAATAGCCTTGCTTCC
lyk4	Forward	GAAGAATGGTTTTGAACGACAAG
	Reverse	AGAAAAGGAAACAGGGAAGTGTC
	Insert	**p745:** AACGTCCGCAATGTGTTATTAAGTTGTC

Table S1. Primers used to confirm plant T-DNA insertions in *fls2, efr, lore,* and *lyk4*.

1.3.2 Barcoded primers for 16S and ITS1 amplification (PCR1) for library preparation on the Illumina MiSeq Platform:

Forward amplification primer description:
Field number (space-delimited), description:
1. 5' Illumina overlap region
2. forward inline barcode (Table S2)
3. gene specific forward primer (Table S3)
TCGTCGGCAGCGTCAGATGTGTATAAGAGACAG NNNNNN LENGTHVARIES

Reverse amplification primer description:
Field number (space-delimited), description:
1. 3' Illumina overlap region
2. inline forward barcode (Table S2)
3. gene specific reverse primer (Table S3)
GTCTCGTGGGCTCGGAGATGTGTATAAGAGACAG NNNNNN LENGTHVARIES

Forward barcode name	Forward barcode sequence	Reverse barcode name	Reverse barcode sequence
t1 forward	GACTAC	t1 reverse	AAGGCC
t2 forward	CTGGTT	t2 reverse	GTCAGG
t3 forward	ACTCGA	t3 reverse	CCTCTT
t4 forward	TGCTGT	t4 reverse	TCGTAG

Table S2. Barcodes used with gene specific amplification primers in Illumina library prep.

Amplicon primer name	Primer sequence
**16S** 799F forward	AACMGGATTAGATACCCKG
**ITS1** forward	CTTGGTCATTTAGAGGAAGTAA
**16S** 1193R reverse	ACGTCATCCCCACCTTCC
**ITS1** reverse	GCTGCGTTCTTCATCGATGC

Table S3. Primers used in gene specific (16S and ITS) amplification for Illumina library preparation. Name includes target and primer name.

1.3.3 Illumina Indexing primers

P5 (forward) indexing primer description:
Field number (space-delimited), description:
1. 5' Illumina adapter
2. i5 index (Table S4)
3. PCR 1 overlap region

AATGATACGGCGACCACCGAGATCTACAC NNNNNNNN TCGTCGGCAGCGTC

P7 (reverse) indexing primer description:
Field number (space-delimited), description:
1. 3' Illumina adapter
2. i7 index (Table S4)
3. PCR 1 overlap region

CAAGCAGAAGACGGCATACGAGAT NNNNNNNN GTCTCGTGGGCTCGG

i5 name	i5 index	i7 name	i7 index
SA501	ATCGTACG	NA701	AACTCTCG
SA502	ACTATCTG	NA702	ACTATGTC
SA503	TAGCGAGT	NA703	AGTAGCGT
SA504	CTGCGTGT	NA704	CAGTGAGT
SA505	TCATCGAG	NA705	CGTACTCA
SA506	CGTGAGTG	NA706	CTACGCAG
SA507	GGATATCT	NA707	GGAGACTA
SA508	GACACCGT	NA708	GTCGCTCG
		NA709	GTCGTAGT
SB501	CTACTATA	NA710	TAGCAGAC
SB502	CGTTACTA	NA711	TCATAGAC
SB503	AGAGTCAC	NA712	TCGCTATA
SB504	TACGAGAC		
SB505	ACGTCTCG	NB701	AAGTCGAG
SB506	TCGACGAG	NB702	ATACTTCG
SB507	GATCGTGT	NB703	AGCTGCTA
SB508	GTCAGATA	NB704	CATAGAGA
		NB705	CGTAGATC
SC501	ACGACGTG	NB706	CTCGTTAC
SC502	ATATACAC	NB707	GCGCACGT
SC503	CGTCGCTA	NB708	GGTACTAT
SC504	CTAGAGCT	NB709	GTATACGC
SC505	GCTCTAGT	NB710	TACGAGCA
SC506	GACACTGA	NB711	TCAGCGTT
SC507	TGCGTACG	NB712	TCGCTACG
SC508	TAGTGTAG		
SD501	AAGCAGCA		
SD502	ACGCGTGA		
SD503	CGATCTAC		
SD504	TGCGTCAC		
SD505	GTCTAGTG		
SD506	CTAGTATG		
SD507	GATAGCGT		
SD508	TCTACACT		

Table S4. Illumina indices used in library amplification.

1.4 PCR recipes and reactions

Ingredient	Volume
HiFi Buffer	3 μL
10 mm dNTPs	0.45 μL
10 uM forward primer	0.45 μL
10 uM reverse primer	0.45 μL
HiFi HotStart enzyme	0.2 μL
PCR water	9.45 μL
Template	1 μL
Total Volume	15 μL

Table S5. PCR1 recipe for 16S and ITS1 library amplification.

Temperature	Time	Step number
95°C	3:00	1
98°C	0:20	2
61°C	0:15	3
72°C	0:15	4
		Repeat steps 2-4 30x
72°C	1:00	5
4°C	hold	6

Table S6. PCR1 cycling for amplification and inline barcoding for 16S and ITS libraries.

Ingredient	Volume
HiFi Buffer	3 μL
10 mm dNTPs	0.45 μL
5 uM i5 Index	1.5 μL
5 uM i7 Index	1.5 μL
HiFi HotStart enzyme	0.2 μL
PCR water	6.35 μL
Template 1	1 μL
Template 2	1 μL
Total Volume	15 μL

Table S7. PCR recipe for Illumina indexing marker gene sequencing.

Temperature	Time	Step number
Temperature Time Step Number 95°C	3:00	1
95°C	0:30	2
55°C	0:30	3
72°C	0:30	4
		Repeat steps 2-4 8x
72°C	5:00	5
4°C	hold	6

Table S8. Library preparation PCR2: PCR cycling for Illumina indexing of PCR1 amplicons.

1.5 Synthetic spike sequences

All spike sequences were carried on a plasmid in *E. coli* TOP10 cells. Spike plasmids were purified using a QIAGEN MiniPrep following manufacturer's instructions. 16S spikes were designed in-house, the spike for ITS1 was designed by Tkacz et al. (2018).

Spike target sequences
Field number (space-delimited), description:
1) Forward primer for co-amplification.
2) Synthetic filler region.
3) Reverse complement of reverse primer for co-amplification.

16S, 799F-1193R: AACCGGATTAGATACCCGG gtgacccccgttcgccttcgatcggctcaccatattaatcttggtggcagacctttagaggattgtgttgctcgactatgaccgactttgtacttcaccgtttgggggagaagcctaccttggccttgcccaccggctgcggtcacggagaagcgagaccgttgacgccgtcagagaaagaatcgagaactggcaaatcgggtgggatagctaactagtggcggctgaccaataggcagtgagaatgcttccgggtccacagccgcttcggttccgttattggtctatacagagctcctggctcaggaactgacacataccccact GGAAGGTGGGGATGACGT

ITS1: (Tkacz et al., 2018) CTTGGTCATTTAGAGGAAGTAA tcccttgtctcctaaaaaacaaagatttactatgcacagaggaacgtctatctaacggttggtatcttgaatgctcggtcccttttgtcattccggattaattcatttccctcattcacaagcttgcgaagtctatattgatatatgaatgcaatctagaagagggcacttaaaattagcagtagttaatattttaaactccatttggtttattcgttacgagactgatt GCATCGATGAAGAACGCAGC

2 SUPPLEMENTARY TABLES AND FIGURES

2.1 Supplementary Figures

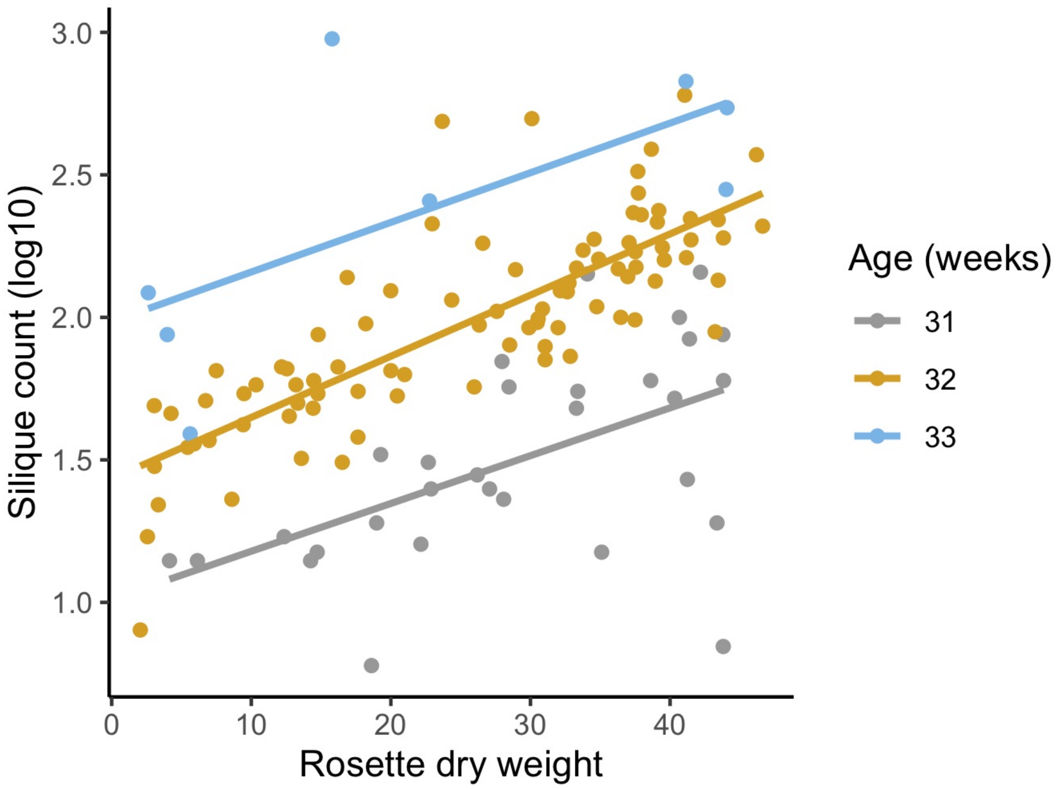

Figure S1. Rosette dry weight significantly correlates with silique count in week 31 and week 32. Pearson correlation of rosette dry weight and silique counts by plant age in weeks. Week 31: n = 30, R = 0.55, p = 0.002; Week 32: n=88, R = 0.81, $p < 0.001$; Week 33: n = 8, R = 0.67, p = 0.07.

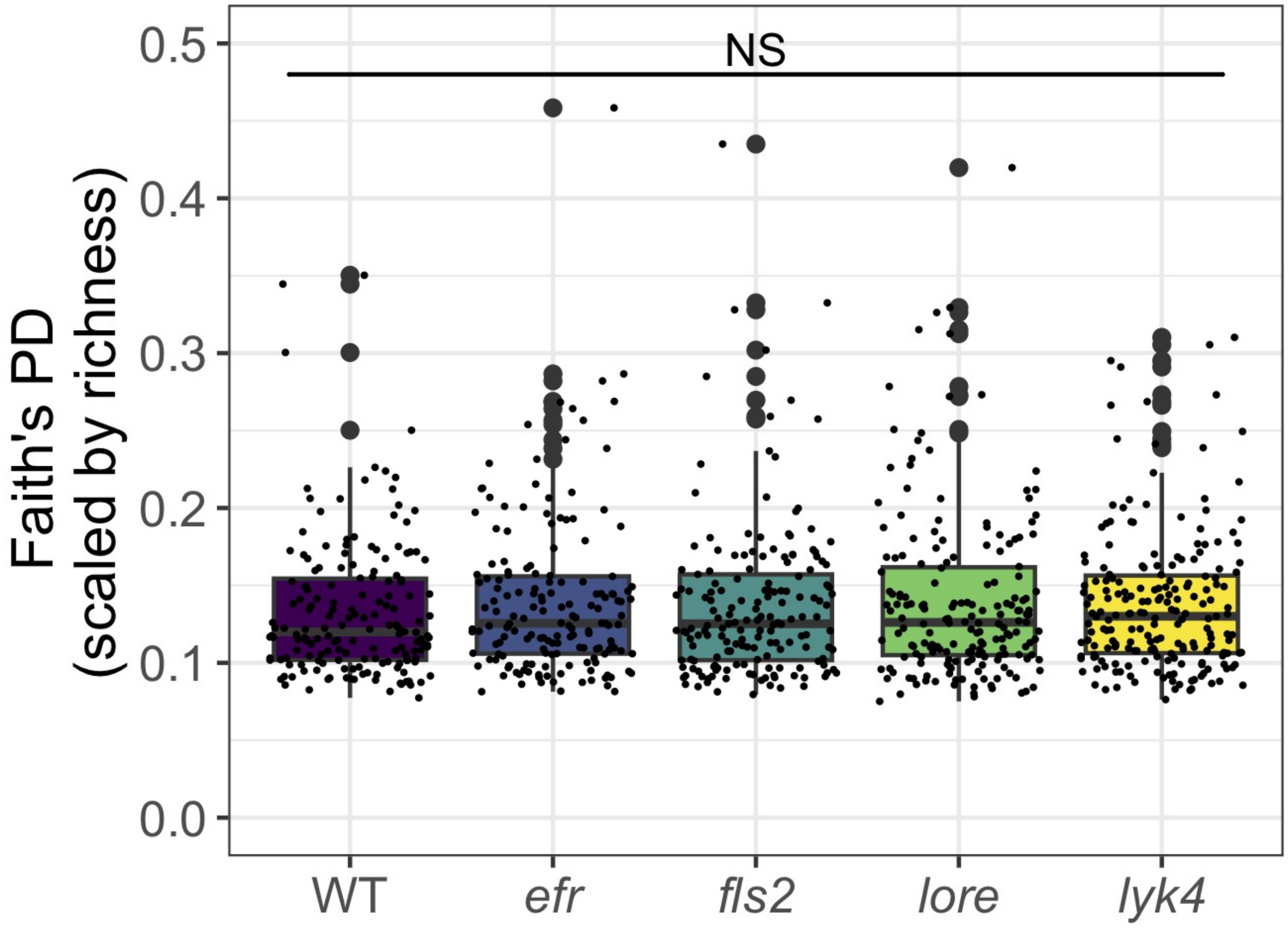

Figure S2. No significant effect of PRR knockout on Faith's Phylogenetic Distance (corrected for species richness) in bacterial microbiomes. Endophytic bacterial communities in PRR knockouts *efr, fls2, lore*, and *lyk4* do not differ in Faith's Phylogenetic Distance compared to wild-type plants (3-way ANOVA, $p > 0.05$, NS = not significant).

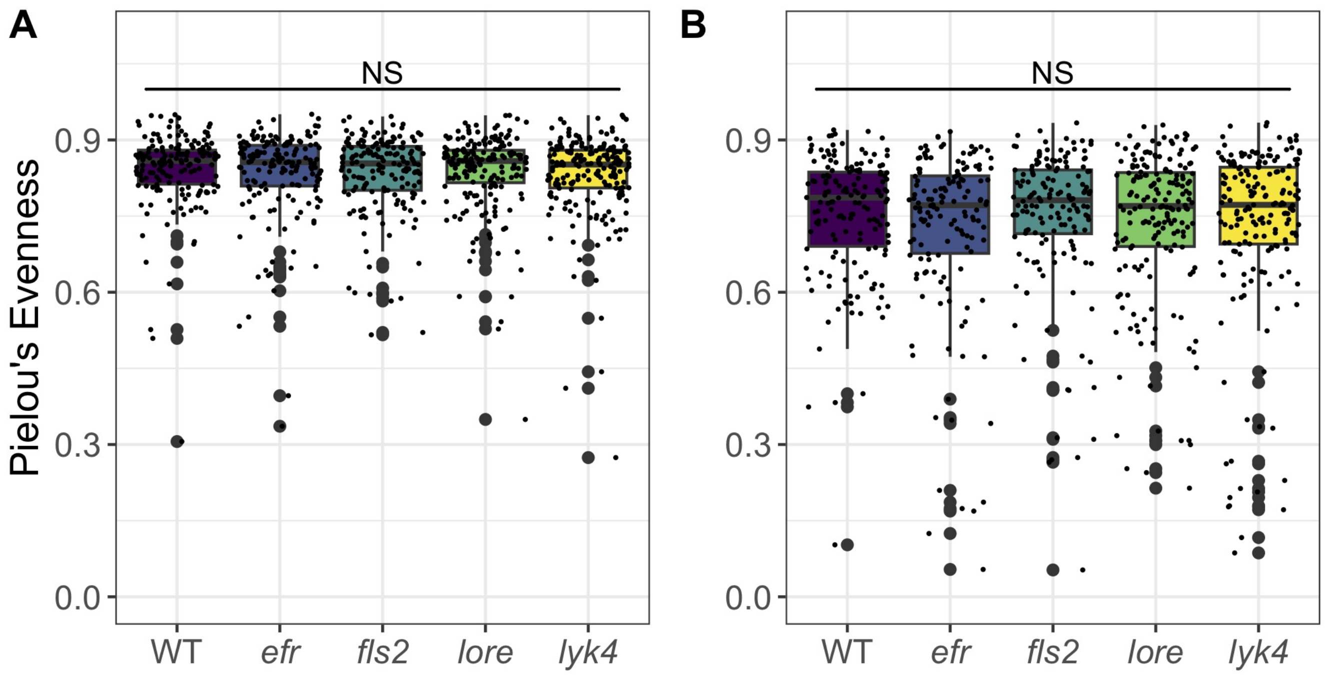

Figure S3. No significant effect of PRR knockout on Pielou's evenness in bacterial or fungal microbiomes. Bacterial (A) or fungal (B) microbiomes in PRR knockouts *efr, fls2, lore*, and *lyk4* do not differ in Pielou's evenness compared to the microbiome associated with wild-type plants (3-way ANOVA, $p > 0.05$, NS = not significant).

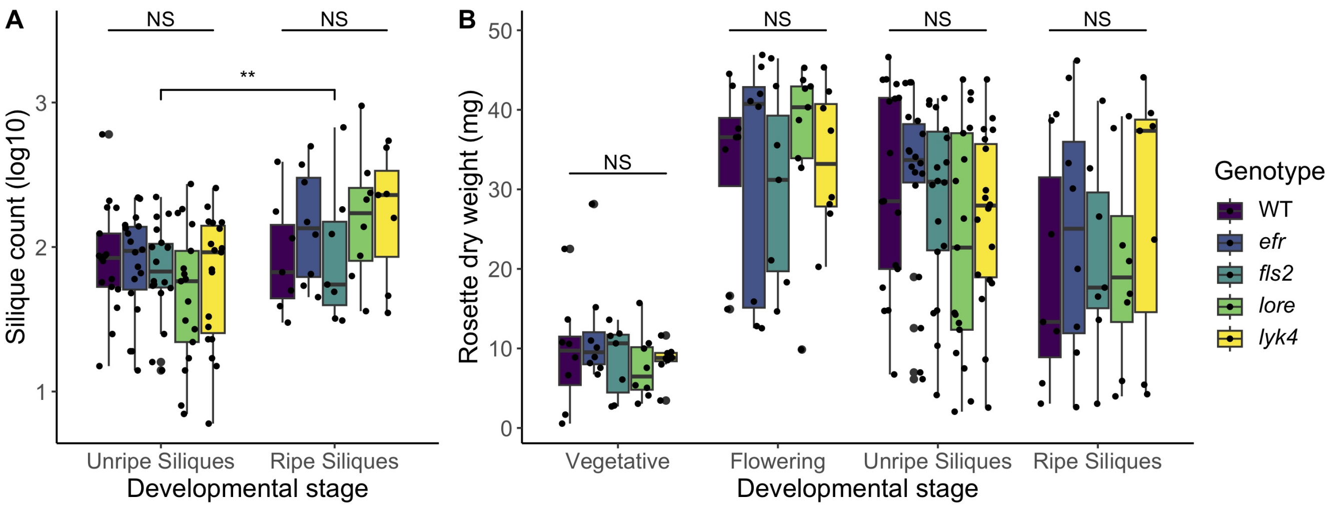

Figure S4. No significant effect of PRR knockout on early plant fitness in the field. (A) Silique counts and (B) rosette dry weight were measured as early fitness indicators. PRR knockouts *efr, fls2, lore*, and *lyk4* do not have significantly different silique counts (Kruskal-Wallis within stage, $p > 0.05$) or rosette dry weight (Kruskal-Wallis within stage, $p > 0.05$) than wild-type plants when grown under field conditions. Sample size per genotype within stage: Vegetative: n = 7-8, Flowering: n = 7-8, Immature Siliques: n = 17-19, Mature Siliques: n = 7-8. Significant differences between genotypes within developmental stage or between developmental stages (all genotypes considered equivalent) according to global Kruskal-Wallis: ** = $p < 0.05$; NS = not significant.

2.2 Supplementary Tables

Diversity Metric	Factor	df	*pseudo*-F	*p*
Shannon Diversity	Tissue	5	21.066	<0.001 *
	Stage	3	4.294	0.007 *
	Genotype	4	0.749	0.565
	Tissue x Stage	10	3.122	<0.001 *
	Tissue x Genotype	20	0.763	0.742
	Stage x Genotype	12	0.517	0.891
	Tissue x Stage x Genotype	40	0.858	0.708
Faith's Phylogenetic Distance (adjusted)	Tissue	5	23.732	<0.001 *
	Stage	3	0.755	0.523
	Genotype	4	0.516	0.749
	Tissue x Stage	10	2.422	0.009 *
	Tissue x Genotype	20	1.067	0.377
	Stage x Genotype	12	0.971	0.458
	Tissue x Stage x Genotype	40	0.71	0.91
Pielou's evenness	Tissue	5	11.522	<0.001 *
	Stage	3	3.962	0.009 *
	Genotype	4	0.464	0.756
	Tissue x Stage	10	3.127	<0.001 *
	Tissue x Genotype	20	0.633	0.889
	Stage x Genotype	12	0.351	0.969
	Tissue x Stage x Genotype	40	1.204	0.185

Table S9. Genotype does not explain α-diversity variation in repeat rarefied bacterial microbiomes. Permutational ANOVA analyses on Shannon diversity, Faith's Phylogenetic Distance adjusted for species richness, and Pielou's Evenness. Variation in endophytic microbiome α-diversity is not explained by genotype as a main effect nor an interaction between genotype and other factors ($p > 0.05$). Tissue, developmental stage and their interaction affect α-diversity ($p < 0.05$). Total samples n=888; genotype by tissue by stage subsets n=3-18.

Diversity Metric	Factor	df	*pseudo*-F	*p*
Shannon Diversity	Tissue	5	41.031	<0.001 *
	Stage	3	10.841	<0.001 *
	Genotype	4	0.603	0.685
	Tissue x Stage	10	3.446	<0.001 *
	Tissue x Genotype	20	0.928	0.551
	Stage x Genotype	12	0.882	0.548
	Tissue x Stage x Genotype	40	1.011	0.445
Pielou's evenness	Tissue	5	46.298	<0.001 *
	Stage	3	2.9	0.038 *
	Genotype	4	1.826	0.104
	Tissue x Stage	10	2.88	0.002 *
	Tissue x Genotype	20	0.769	0.736
	Stage x Genotype	12	0.83	0.613
	Tissue x Stage x Genotype	40	0.948	0.566

Table S10. Genotype does not explain α-diversity variation in repeat rarefied fungal microbiomes. Permutational ANOVA analyses on Shannon diversity and Pielou's Evenness. Variation in endophytic ITS1 microbiome α-diversity is not explained by genotype as a main effect nor an interaction between genotype and other factors ($p > 0.05$). Tissue, developmental stage and their interaction affect α-diversity ($p < 0.05$). Total samples n=816; genotype by tissue by stage subsets: n=2-17.

Diversity Metric	Factor	df	R^2	*pseudo*-F	*p*
Bray-Curtis	MiSeq Run	5	0.016	3.674	0.001 *
	Tissue	5	0.183	43.159	0.001 *
	Stage	3	0.031	12.122	0.001 *
	Genotype	4	0.003	1.003	0.458
	MiSeq Run:Plate	9	0.019	2.451	0.001 *
	Tissue x Stage	10	0.031	3.596	0.001 *
	Tissue x Genotype	20	0.016	0.954	0.736
	Stage x Genotype	12	0.010	0.934	0.783
	Tissue x Stage x Genotype	40	0.031	0.915	0.972
Jaccard	MiSeq Run	5	0.013	2.694	0.001 *
	Tissue	5	0.119	25.229	0.001 *
	Stage	3	0.023	8.041	0.001 *
	Genotype	4	0.004	1.015	0.414
	MiSeq Run:Plate	9	0.017	1.973	0.001 *
	Tissue x Stage	10	0.028	3.011	0.001 *
	Tissue x Genotype	20	0.018	0.974	0.734
	Stage x Genotype	12	0.011	0.947	0.839
	Tissue x Stage x Genotype	40	0.036	0.947	0.97
Weighted UniFrac	MiSeq Run	5	0.026	6.110	0.001 *
	Tissue	5	0.327	77.960	0.001 *
	Stage	3	0.071	28.423	0.001 *
	Genotype	4	0.003	0.771	0.714
	MiSeq Run:Plate	9	0.018	2.446	0.001 *
	Tissue x Stage	10	0.028	3.382	0.001 *
	Tissue x Genotype	20	0.012	0.702	0.981
	Stage x Genotype	12	0.009	0.889	0.696
	Tissue x Stage x Genotype	40	0.030	0.885	0.824

Table S11. Genotype does not explain β-diversity variation in rarefied 16S core communities. PERMANOVA analyses on β-diveristy distance matrices generated with Bray-Curtis, Jaccard and Weighted Unifrac β-diversity meterics on rarefied data sets. ASVs were included in this analysis if present at 1% relative abundance in 20% of samples in at least one tissue by stage subset (Core B). Genotype does not explain 16S core community β-diversity variation as a main effect nor interact with other factors. Statistically significant factors are denoted by asterisks and include tissue, developmental stage and their interaction (fixed effects), MiSeq run and PCR plate nested within MiSeq run (random effects). n=3-18 for each genotype by tissue by stage subset, with n=888 total samples.

Diversity Metric	Factor	df	R^2	*pseudo*-F	*p*
Robust CLR	MiSeq Run	5	0.012	2.600	0.001 *
	Tissue	5	0.121	26.385	0.001 *
	Stage	3	0.017	6.301	0.001 *
	Genotype	4	0.004	1.049	0.266
	MiSeq Run:Plate	9	0.012	1.429	0.001 *
	Tissue x Stage	10	0.027	2.952	0.001 *
	Tissue x Genotype	20	0.018	0.958	0.801
	Stage x Genotype	12	0.012	1.081	0.101
	Tissue x Stage x Genotype	40	0.036	0.989	0.58
ALR	MiSeq Run	5	0.014	2.477	0.001 *
	Tissue	5	0.176	31.046	0.001 *
	Stage	3	0.026	7.592	0.001 *
	Genotype	4	0.005	1.095	0.204
	MiSeq Run:Plate	9	0.017	1.657	0.001 *
	Tissue x Stage	10	0.033	2.904	0.001 *
	Tissue x Genotype	20	0.022	0.955	0.761
	Stage x Genotype	12	0.015	1.086	0.166
	Tissue x Stage x Genotype	40	0.043	0.955	0.834

Table S12. Genotype does not explain β-diversity variation in log-transformed 16S core communities. PERMANOVA analyses on Euclidean distances between robust CLR or ALR transformed core communities. ASVs were included in this analysis if present at 1% relative abundance in 20% of samples of at least one tissue by stage subset (Core B). Genotype does not explain 16S core community β-diversity variation as a main effect nor does genotype interact with other factors. Statistically significant factors are denoted by asterisks and include tissue, developmental stage and their interaction (fixed effects), MiSeq run and PCR plate nested within MiSeq run (random effects). CLR: total samples *n*= 917, tissue by genotype by stage subsets, *n*=3-19. ALR: total samples *n*=684, tissue by genotype by stage subsets, *n*= 2-15.

Diversity Metric	Factor	df	R^2	*pseudo-F*	*p*
Bray-Curtis	MiSeq Run	2	0.012	6.309	0.001 *
	Tissue	5	0.195	42.710	0.001 *
	Stage	3	0.027	9.947	0.001 *
	Genotype	4	0.005	1.349	0.031 *
	MiSeq Run:Plate	11	0.020	1.972	0.001 *
	Tissue x Stage	10	0.033	3.670	0.001 *
	Tissue x Genotype	20	0.017	0.915	0.862
	Stage x Genotype	12	0.013	1.182	0.047 *
	Tissue x Stage x Genotype	40	0.033	0.912	0.947
Jaccard	MiSeq Run	2	0.008	4.124	0.001 *
	Tissue	5	0.130	25.489	0.001 *
	Stage	3	0.020	6.461	0.001 *
	Genotype	4	0.005	1.218	0.034 *
	MiSeq Run:Plate	11	0.018	1.619	0.001 *
	Tissue x Stage	10	0.029	2.819	0.001 *
	Tissue x Genotype	20	0.019	0.954	0.803
	Stage x Genotype	12	0.014	1.114	0.052
	Tissue x Stage x Genotype	40	0.038	0.932	0.982

Table S13. Genotype explains a small fraction of β-diversity variation of rarefied ITS1 core communities. PERMANOVA analyses on β-diversity distance matrices generated by Bray-Curtis and Jaccard distances on rarefied data sets ASVs were included in this analysis if present at 1% relative abundance in 20% of samples of at least one tissue and stage subset (Core B). Genotype explains a small fraction (0.5%) of ITS1 core community variation as a main effect and significantly interacts with developmental stage in Bray-Curtis community variation. The genotype-stage interaction is marginal using the Jaccard Index. Statistically significant factors and/or interactions are denoted by asterisks. n=2-17 for each genotype by tissue by stage subset, with n=816 total samples.

Diversity Metric	Factor	df	R^2	*pseudo-F*	*p*
Robust CLR	MiSeq Run	2	0.006	3.328	0.001 *
	Tissue	5	0.117	24.008	0.001 *
	Stage	3	0.024	8.120	0.001 *
	Genotype	4	0.005	1.187	0.077
	MiSeq Run:Plate	11	0.015	1.434	0.001 *
	Tissue x Stage	10	0.037	3.842	0.001 *
	Tissue x Genotype	20	0.018	0.947	0.833
	Stage x Genotype	12	0.013	1.121	0.057
	Tissue x Stage x Genotype	40	0.036	0.937	0.962
ALR	MiSeq Run	2	0.012	3.939	0.001 *
	Tissue	5	0.150	19.750	0.001 *
	Stage	3	0.031	6.895	0.001 *
	Genotype	4	0.007	1.118	0.175
	MiSeq Run:Plate	11	0.022	1.328	0.001 *
	Tissue x Stage	10	0.034	2.227	0.001 *
	Tissue x Genotype	20	0.027	0.896	0.961
	Stage x Genotype	12	0.017	0.932	0.82
	Tissue x Stage x Genotype	36	0.047	0.870	1

Table S14. Genotype does not explain β-diversity variation of log-transformed ITS1 core communities. PERMANOVA analyses on the Euclidean distance between robust-CLR or ALR transformed communities. ASVs were included in this analysis if present at 1% relative abundance in 20% of samples of at least one tissue and stage subset (Core B). Genotype is not statistically significant after robust CLR transformation or ALR transformation (PERMANOVA, $p > 0.05$). Statistically significant factors and/or interactions are denoted by asterisks. CLR: total samples n=855, tissue by genotype by stage subsets, n=3-17. ALR: total samples n=534, tissue by genotype by stage subsets, n= 1-17.

Marker	Subset type	Subset name	df	*pseudo*-F	*p*
16S	Overall (none)	Overall (none)	4	0.873	0.469
	Within tissues	Roots	4	0.827	0.508
		Rosettes	4	1.410	0.251
		Stems	4	0.470	0.755
		Cauline leaves	4	0.305	0.872
		Siliques	4	0.328	0.849
		Flowers	4	0.639	0.631
	Within stage	Vegetative	4	1.174	0.322
		Flowering	4	0.205	0.931
		Unripe siliques	4	1.497	0.184
		Ripe siliques	4	0.730	0.553
ITS	Overall (none)	Overall (none)	4	0.649	0.616
	Within tissues	Roots	4	1.470	0.229
		Rosettes	4	1.538	0.193
		Stems	4	0.298	0.874
		Cauline leaves	4	0.448	0.791
		Siliques	4	0.080	0.986
		Flowers	4	0.551	0.695
	Within stage	Vegetative	4	0.330	0.85
		Flowering	4	0.197	0.941
		Unripe siliques	4	0.395	0.805
		Ripe siliques	4	0.635	0.636

Table S15. Microbiome β-diversity dispersions are not different in PRR knockout genotypes compared to wild-type plants. Multivariate homogeneity of group dispersions (PERMDISP2) on Bray-Curtis distances of core 16S and ITS1 microbiomes (Core B) reveals that wild-type and mutant genotypes do not have significantly different variability. The effect of genotype on group dispersions was tested in the overall data set and within tissue, developmental stage, and tissue by stage subsets. This effect was additionally tested using the Jaccard distance, other cores, and with a minimally filtered ASV set (non-core). Similar results were obtained in all analyses (data not shown). Bacteria: overall n=166-200; within tissue n=13-41; within stage *11-98*; genotype by tissue by stage subset n= 3-18. Fungi: overall n= 143-183; within tissue n=21-79; within stage *13-92*; genotype by tissue by stage subset n=2-17.

Marker	Factor	df	*pseudo*-F	*p*
16S	Stage	2	6.969	0.001 *
	Genotype	4	0.609	0.666
	Stage x Genotype	8	0.824	0.590
ITS	Stage	2	7.969	< 0.001 *
	Genotype	4	1.249	0.299
	Stage x Genotype	8	1.127	0.357

Table S16. Within-individual tissue specificity of bacterial and fungal microbiomes is affected by developmental stage but not PRR knockout. The results of permutational ANOVAs testing the effect of developmental stage and genotype on mean Bray-Curtis distance of aerial tissues to the individual plant median, a measure of tissue specificity. Asterisks denote significant results. Only samples from plants with all tissues retained were considered for analysis. Sample size for Stage x Genotype subsets: Bacteria: n=2-13, Fungi: n=2-12.

Marker	Factor	df	*pseudo*-F	*p*
16S	Tissue	5	59.885	<0.001 *
	Stage	3	2.084	0.101
	Genotype	4	1.234	0.295
	Tissue x Stage	10	3.166	0.001 *
	Tissue x Genotype	20	1.388	0.121
	Stage x Genotype	12	1.374	0.174
	Tissue x Stage x Genotype	40	1.027	0.428
ITS	Tissue	5	17.012	<0.001 *
	Stage	3	5.428	0.001 *
	Genotype	4	0.996	0.409
	Tissue x Stage	10	1.795	0.059 .
	Tissue x Genotype	20	0.906	0.580
	Stage x Genotype	12	0.597	0.845
	Tissue x Stage x Genotype	36	0.835	0.741

Table S17. Single PRR knockout does not affect microbial load. ANOVA table of factors tested for an impact on microbial load variation. Relative microbial load was determined by comparing the ratio of spike-in copy number to marker gene copy number, and $\log_{10}$ transformed for analysis. Tissue and developmental stage affect microbial load, but PRR knockout genotype does not. Bacteria: total samples n=686; genotype by tissue by stage subsets n=2-15. Fungi: total samples n=543; genotype by tissue by stage subsets: n=2-17.

REFERENCES

Rohland, N. and Reich, D. (2012). Cost-effective, high-throughput DNA sequencing libraries for multiplexed target capture. *Genome Research* 22, 939–946. doi:10.1101/gr.128124.111

Tkacz, A., Hortala, M., and Poole, P. S. (2018). Absolute quantitation of microbiota abundance in environmental samples. *Microbiome* 6, 1–13. doi:10.1186/s40168-018-0491-7
